# Highly durable organic electrode for sodium-ion batteries via a stabilized α-C radical intermediate

**DOI:** 10.1038/ncomms13318

**Published:** 2016-11-07

**Authors:** Shaofei Wu, Wenxi Wang, Minchan Li, Lujie Cao, Fucong Lyu, Mingyang Yang, Zhenyu Wang, Yang Shi, Bo Nan, Sicen Yu, Zhifang Sun, Yao Liu, Zhouguang Lu

**Affiliations:** 1 Department of Materials Science and Engineering, South University of Science and Technology of China, Shenzhen 518055, China

## Abstract

It is a challenge to prepare organic electrodes for sodium-ion batteries with long cycle life and high capacity. The highly reactive radical intermediates generated during the sodiation/desodiation process could be a critical issue because of undesired side reactions. Here we present durable electrodes with a stabilized α-C radical intermediate. Through the resonance effect as well as steric effects, the excessive reactivity of the unpaired electron is successfully suppressed, thus developing an electrode with stable cycling for over 2,000 cycles with 96.8% capacity retention. In addition, the α-radical demonstrates reversible transformation between three states: C=C; α-C·radical; and α-C^−^ anion. Such transformation provides additional Na^+^ storage equal to more than 0.83 Na^+^ insertion per α-C radical for the electrodes. The strategy of intermediate radical stabilization could be enlightening in the design of organic electrodes with enhanced cycling life and energy storage capability.

## Abstract (web summary)

Reactive radical intermediates can be a problem leading to fast degradation of organic electrodes in sodium-ion batteries. Here, the authors show a stabilized α-C radical displaying reversible transformations between C=C, α-C radical and α-C anionic states to facilitate long cycling and improved capacity.

## Graphical abstract

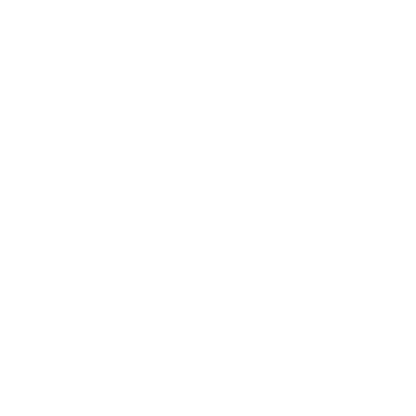

The large-scale production of lithium-ion batteries for vehicle applications may markedly increase the cost of lithium resources in the foreseeable future since lithium reserves are limited and located in relatively few geographical areas^1^,^2^,^3^. Rechargeable sodium-ion batteries (NIBs) have attracted increasing research interest as a promising alternative to lithium-ion batteries (LIBs) due to the natural abundance and low cost of sodium resources^4^,^5^. Irrespective of the great success of LIBs, the application of analogous materials such as intercalation-type inorganic^6^,^7^, carbonaceous material^8^,^9^,^10^,^11^ and alloy-based anodes^12^,^13^,^14^,^15^ in NIBs have been rarely feasible, primarily due to the poor kinetics of the Na^+^ insertion/desertion reaction caused by the relatively larger ionic radius (102 pm) of Na^+^ versus Li^+^ (76 pm)^16^,^17^,^18^. Organic materials have structural flexibility because of their more flexible structures compared with inorganic compounds, which could provide high mobility of Na^+^ ions in the organic electrodes^19^,^20^. Nevertheless, up until now, only a few kinds of organic materials, including quinones^21^,^22^, polyimides^23^, polymeric Schiff bases^24^ and carboxylates^25^,^26^,^27^ have been developed for NIBs, but even most of them still suffered from poor cycle stability or low specific capacity^19^,^22^. To fundamentally address these problems, the free radical intermediates generated during the Na^+^ insertion/extraction process should be taken into account. Accordingly, the reduction of unsaturated bonds such as C=C, C=N and C=O develops radical intermediates containing negative charge mainly on the N and O atoms, and an unpaired electron prominently on the carbon atom^28^. Unfortunately, such kinds of organic radical intermediates are generally unstable and could rapidly transform to inactive compounds by reacting with other molecules in the electrolyte or in between the radicals, which eventually leads to irreversibility of the redox process and thus results in disappointing rechargeability (Fig. 1a)^29^,^30^. This phenomenon has long been investigated in the redox behaviour of carbonyl compounds. For example, the tri-carbonyl-based material, dichloroisocyanuric acid (DCCA) (Fig. 1b), was found to be irreversible due to the formation of inactive precipitates^31^. Therefore, it is highly desirable to stabilize the organic radical intermediates generated during charge and discharge to improve the reversibility of the organic electrodes for energy storage.

A general solution to stabilize the intermediate radicals is incorporating pairs of unsaturated groups into the conjugated compound, which could lead to the formation of a reversible breaking/reformation of double bonds through an unpaired electron internal-consumption mechanism^21^,^22^,^32^. Other effective methods include crosslinking and increasing formula weight that could decrease the solubility of the molecular radicals^23^,^33^. Recently, organic radical compounds in the oxidative state, including nitroxyl^34^, phenoxyl^35^,^36^ and hydrazyl^37^ display excellent cycling stability for energy storage^38^,^39^,^40^. In addition, a wide variety of commercially available organic radical compounds show decent stability in both solid and solution states, and could be used as a radical trapper^41^. These unpaired electrons show less inter-molecule reactivity and are quite stable generally because of their naturally robust structures and sufficient conjugate action with the adjacent electrons^42^,^43^. According to kinetic chemistry and transition state theory^44^, the electron resonance effect drops the reaction potential energy of the radical, while the hindrance effect improves the dimerization reaction energy barrier of the transition state due to an increasing repelling force as depicted in Supplementary Fig. 1. As a result, the expanded molecular reaction potential barrier between the radical and the transition state significantly restricts the combination rate of radical intermediates. Therefore, the disappearance of the unpaired electrons arising from irreversible inter-molecular chemical bond formation could be largely suppressed by an electronic resonance effect and steric hindrance effect of the substitution groups^45^,^46^.

Herein, we display the construction of a highly reversible and stable tri-carbonyl-based electrode by imposing restrictions on the reactivity of radical intermediates. The β-ketoenamine-linked compound is famous for its reversible proton transfers (tautomerism) between quinoidal and enol forms, and can be used for optical switches^47^, gas storage^48^ and energy storage^49^,^50^,^51^,^52^. The highly oxidative isomer could be a potential candidate for electrode material. It has been shown that tris *N*-salicylidenearylamines maintain its highly oxidative form in both solution and solid states^53^. The tri-β-ketoenamine-linked compounds, TSAA (tris *N*-salicylideneanthramine) and TSAQ (tris *N*-salicylideneanthraquinoylamine) (Fig. 2) are chosen as candidates for NIB electrodes. The electron reduction of C=O leads to the breakage of the tri-β-ketoamine system, forming a benzyl radical (α-C radical) intermediate, which is stabilized by the resonance effect of the neighbouring groups (for example, Ar–NH–), as well as the steric hindrance effect of the rigid structure. The TSAQ electrode shows excellent reversible electro-transformation between C=O and C–O–Na and delivers no obvious capacity degradation after more than 2,500 cycles at a high current density of 1 A g^−1^. Furthermore, the generated α-C radical shows the reversible Na^+^ insertion/extraction at low potential (0.3 V, versus Na^+^/Na), which makes the material a promising NIB anode with a high capacity (370 mAh g^−1^ at 50 mA g^−1^) and stable cycle life. The results show that a stabilized radical intermediate could facilitate high reversibility of the organic electrode, with the radical providing the electrode with more redox centres, and thus an improved capacity.

## Results

### Physical characterization

The normal tri-carbonyl compound DCCA was utilized as a comparison due to the similar tri-keto structure while lacking of the extended dual action effect and steric effect of aromatic substituent. TSAA and TSAQ were easily synthesized by the simple metallic catalyst-free condensation reaction from 1,3,5-triformylphloroglucinol and 2-aminoanthracene/2-aminoanthraquinone, respectively (Supplementary Fig. 2). The nuclear magnetic resonance (NMR) chemical structure analysis could be seen in Supplementary Figs 3–6. The *π*-conjugated designs of the TSAA and TSAQ are expected to be beneficial to electron transport facilitating high rate capability at high current density. Simultaneously, the *π*-conjugated system is also helpful to construct a layer-by-layer molecular arrangement through the intermolecular interactions (for example, *π*−*π* or C–H···*π* interactions), which is favourable to form a fast ion diffusion channel between two layers^19^. The scanning electron microscopy (SEM), transmission electron microscopy (TEM), X-ray diffraction and Brunauer–Emmett–Teller theory methods have been used to investigated the morphology of TSAA and TSAQ (Supplementary Fig. 7). SEM images suggests a foam-like structure consisting of nanosheets and nanowires. X-ray diffraction pattern exhibits diffraction features with broad peaks at ∼2*θ*°=28.1° and 26.4°, corresponding to a *d*-space of about 0.32 and 0.34 nm for the TSAA and TSAQ, respectively. The high-resolution TEM images, Brunauer–Emmett–Teller theory and pore size distribution results illustrate abundant porous structure for both TSAA and TSAQ, which is favourable for fast insertion/extraction of sodium-ion where the electrochemical reaction happens. The thermal gravimetric analysis (TGA) study (Supplementary Fig. 8) in N_2_ atmosphere suggests superior thermal stability, with only 10 wt% loss up to 400 °C. Moreover, the strong inter-/intramolecular interaction (for example, *π*−*π*, C–H···*π* interactions and hydrogen bond) among the layered structure and relatively large molecular weight endow both the materials and theirs charged states with poor solubility in solvents like ethylene carbonate (EC), dimethyl carbonate (DMC), dimethylsulphoxide, *N*-methyl-2-pyrrolidone (NMP), CHCl_3_ and ethanol. This property is significant in improving the cycling stability of organic electrodes in organic electrolytes.

### Electrochemical properties of the electrodes

**Figure 3:**
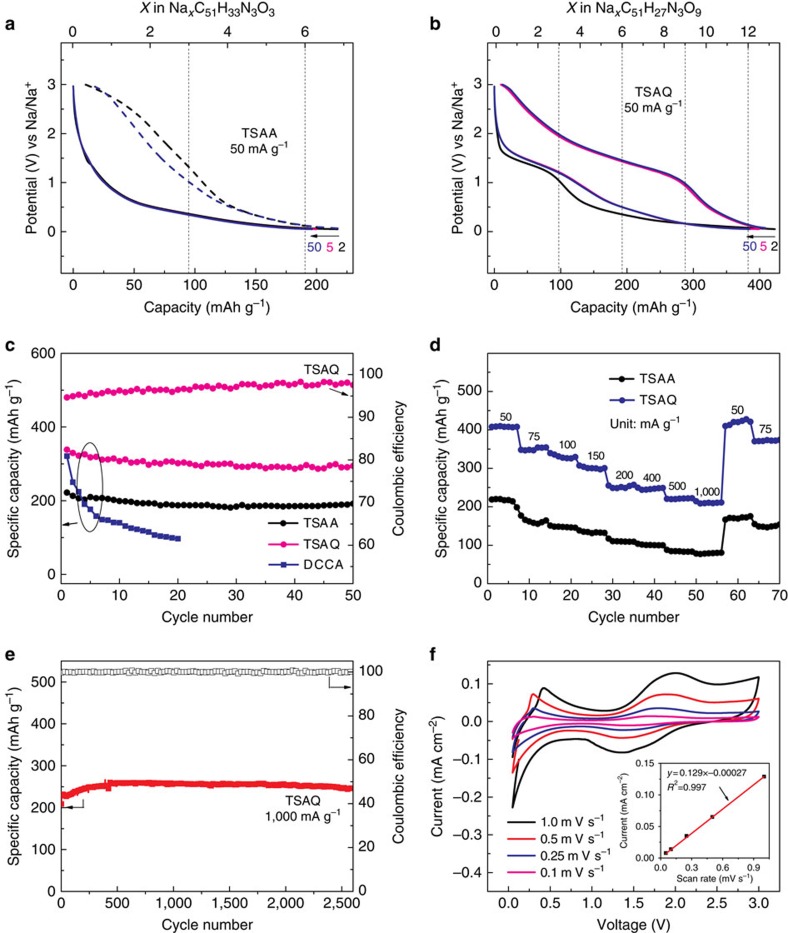
Electrochemical properties of the electrodes. The second (black), fifth (magenta)and fiftieth (blue) galvanostatic discharge–charge curves of the TSAA (**a**) and TSAQ (**b**) at a current density of 50 mA g^−1^. (**c**) Cycle performances at a current density of 100 mA g^−1^ in the voltage range of 3–0.05 V versus Na/Na^+^; (**d**) rate performance of TSAA and TSAQ electrodes at various current densities. (**e**) Cycle performance and the corresponding coulombic efficiency of TSAQ at a current density of 1,000 mA g^−1^ in the voltage range of 3–0.05 V versus Na^+^/Na up to more than 2,500 cycles. (**f**) CV curves of TSAQ at a scanning rate of 1.0, 0.5, 0.25 and 0.1 mV s^−1^, and the relation between scanning rate and peak current (insert).

Figure 3a,b shows the galvanostatic discharge/charge voltage profiles of the second, fifth and fiftieth cycles of the TSAQ and TSAA, respectively, at a current density of 50 mA g^−1^ in the voltage range of 0.05–3 V (versus Na^+^/Na). The initial discharge/discharge curves of these two samples are shown in Supplementary Fig. 9a,b. The first, second, fifth and tenth cycles of the cyclic voltammogram of these two samples are shown in Supplementary Fig. 9c,d. It is apparent that the cyclic voltammetry (CV) curve of the first anodic process is quite different from those of subsequent cycles. The well-defined peaks centred at about 0.5 V (versus Na^+^/Na) only appear in the first CV cycle could be ascribed to the occurrence of some irreversible reactions via the formation of solid electrolyte interface (SEI) film, similar phenomenon generally observed in the other electrodes^54^,^55^. The same phenomenon could also be seen from the exclusive plateau ranging from 0.8 to 0.4 V of the TSAA and TSAQ first discharge curve. The coulombic efficiency of the initial cycle of the TSAA and TSAQ sample is calculated about 56% and 51%, respectively. The large irreversibility of the initial cycle was mainly attributed to the formation of a thick SEI on the surface of the organic electrode materials at the potential of about 0.5 V. From the second cycle onwards, the Coulombic efficiencies witness an upward trend and remain a high efficiency over 96% after the fifth cycle, indicating an enhancement of the reversibility of sodium insertion and extraction process. For the TSAA sample, the voltage profiles become slopping ranging from 0.9 to 0.05 V delivering a specific capacity of about 200 mAh g^−1^. While the TSAQ electrode displays two obvious plateaus with one ranging from about 1.5 to 0.5 V representing a capacity of 195 mAh g^−1^ and another ranging from 0.5 to 0.05 V with a capacity of 200 mAh g^−1^, thus an overall capacity of about 395 mAh g^−1^ was obtained. As a result, TSAA and TSAQ electrodes show highly reversible sodium insertion/extraction at an average voltage of 0.3 and 0.6 V, respectively. The slopping range below 0.5 V linked to the sodium-ion insertion into radical also displays apparent polarization but much less than that of the carbonyls (Supplementary Fig. 10). After deducting the capacity contribution from the super P (Supplementary Fig. 11), the TSAA and TSAQ electrode delivers a real reversible specific capacity of about 172 and 366 mAh g^−1^, corresponding to about 5.5 and 11.5 sodium-ion per molecule, respectively.

Figure 3c shows the cycling performance of the organic electrodes at a current density of 100 mA g^−1^. The DCCA electrode demonstrates a very poor cycle performance with <30% retention of the initial capacity after 20 cycles, which implies highly irreversible character of the redox centre, that is, C=O in the normal DCCA organic skeleton without the stabilization effects. Such an irreversible nature could be ascribed to the formation of inactive compound such as dimer due to the excessive reactive tendency of the radical intermediates. Moreover, the slight dissolution of the radical intermediate of DCCA in the electrolyte makes the dimer reaction even more serious due to greater reaction rate in the solution state than that in the solid state. Therefore, the active materials in the DCCA electrode may finally transform to the inactive materials in high rate causing extreme capacity degradation in several cycles. In a good contrast, the TSAA and TSAQ electrodes show a highly reversible capacity of 187 and 320 mAh g^−1^, respectively, and the capacity retentions are higher than 95% after 50 cycles for both electrodes. The very limited dissolubility (Supplementary Figs 12 and 13) as well as restricted reactive tendency of the radical intermediates successfully prevent the formation of inactive participate as that of DCAA facilitating superior cycling stability (see detailed discussion in Supplementary Note 1). The β-ketoamine structure has largely suppressed reactive tendency of the carbon free radical and thereby guaranteed a comparably stable radical intermediates even in the solution state.

According to the results above, the core structure, tri-β-ketoamine, displayed a highly durable electrochemical reaction, as well as a high capacity. As can be seen from Fig. 3d, in addition to superior cycle stability, the TSAQ sample also exhibited excellent rate capability, yielding capacities of 405, 355, 330, 300, 265, 250, 240 and 220 mAh g^−1^ at the current densities of 50, 75, 100, 150, 200, 400, 500 and 1,000 mA g^−1^, respectively. While the TSAA delivered capacities of 215, 185, 170, 155, 130, 110 and 95 mAh g^−1^ at the same current densities, respectively. The specific capacity of the TSAA is less than that of the TSAQ, indicating that the redox-active group modification of the side rigid substitution improves the energy storage capability. However, when the current was recovered to 50 mA g^−1^, both the TSAQ and TSAA samples restored more than 90% of their initial capacities. This means that cycling at high current density hardly damaged the structure of the tri-β-ketoamine electrodes, given that the capacity is restored within the applied current density. To the best of our knowledge, very few NIB electrodes could sustain such high current densities of 1,000 mA g^−1^. Furthermore, when cycled at the high current rate of 1 A g^−1^, the TSAQ displayed an increasing capacity at the beginning from about 218 to the saturated value of 258 mAh g^−1^ after 500 cycles, and the capacity was even higher than 250 mAh g^−1^ after 2,000 cycles (Fig. 3e), implying ultra-stable cycling for Na^+^ insertion/extraction. Electrochemical impedance spectroscopy (Supplementary Fig. 14) was conducted to study the electronic conductivity and sodium-ion diffusion in the TSAQ electrode. It is obvious that a semicircle at medium frequency region. In this plot, the semicircle, corresponding to sodium ions passing through the SEI film and charge transfer between electrolyte and active material, is rigidly consistent with the simulated impedance data. Before cycle, the resistance value of *R*_ele_ and *R*_ct_ is around 12 and 107 Ω, respectively. After activation for 100 cycles, the resistance value of *R*_ele_ and *R*_ct_ decreases to around 21 and 22 Ω, hence indicating faster charge transport and better high rate performance. As also can be seen from the cyclic voltammograms of TSAQ at different scanning rates (Fig. 3f), the peak current increased linearly with the sweep rate, indicating that sodium-ion diffusion is not rate-determining^28^. Therefore, the organic electrodes based on tri-β-ketoamine exhibited a good combination of high capacity, stable cycle life and excellent rate capability.

## Discussion

**Figure 4:**
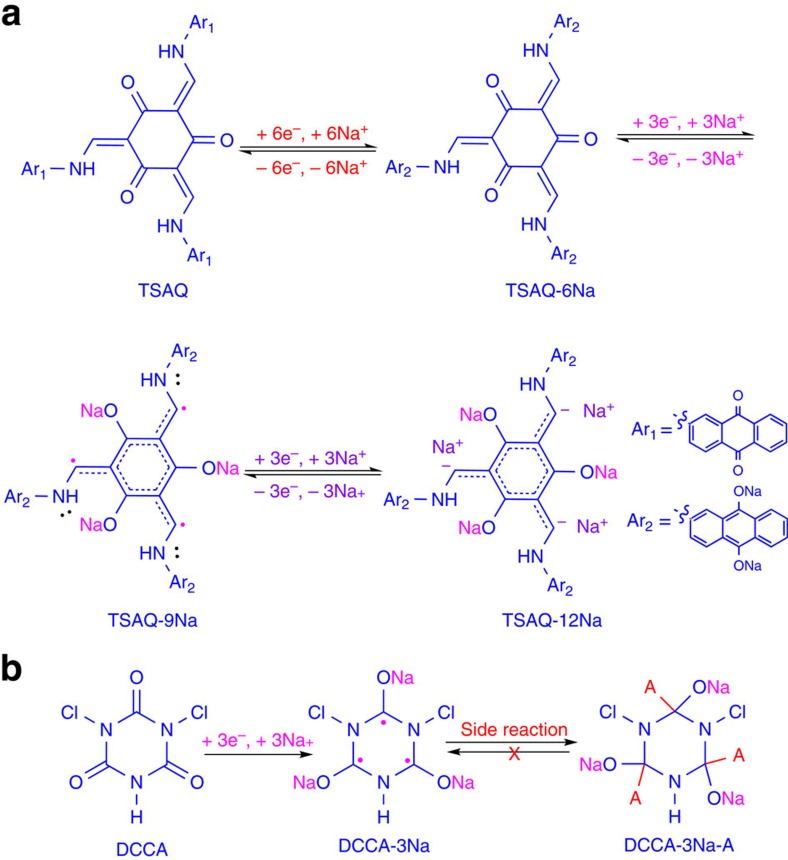
Sodium-ion insertion/extraction mechanism of the electrodes. (**a**) The reversible electro-chemical process of TSAQ electrode and (**b**) the generation of inactive material of DCCA electrode. ‘A' refers to groups reacting with the radical intermediate.

The supposed mechanism of the sodiation and desodiation of TSAQ was shown in Fig. 4a. First, the reduction of quinone corresponding to the sloping plateau at about 1.2 V results in the formation of sodium enolate (TSAQ-6Na). Then three-electron reduction of β-keto at around 0.7 V leads to the breakage of the 12-*π*-electron Hückel system and the formation of a 9-*π*-electron aromatic Hückel ring, which could further transform to benzyl radical intermediates (α-C· radical) stabilized by the dual action of enamine and the generated benzene ring (TSAQ-9Na). The α-C· radical intermediate is expected to be electrophilic as the uncharged state *n*-type radical polymers, and such a property helps to appeal additional electrons (α-C^−^ anion) and coordinate Na^+^, thus giving TSAQ-12Na. All of the processes show excellent reversibility thus resulting in stable cycling. As a contrast, the three-electron reduction of DCCA electrode also gives a C· radical intermediate (Fig. 4b), that is, DCCA-3Na. However, DCCA-3Na immediately transfers to electro-inactive DCCA-3Na-A because of the side reaction within radical compound or with electrolyte. Compared with TSAA-9Na, the intermediate DCAA-3Na shows obviously weaker stabilization effects from the conjugate action, and also the DCAA-3Na displays almost a flexible structure that poses an inferior restrict action on the reactive radical. As a consequence, the DCCA electrode demonstrates very poor cycle performance.

**Figure 5:**
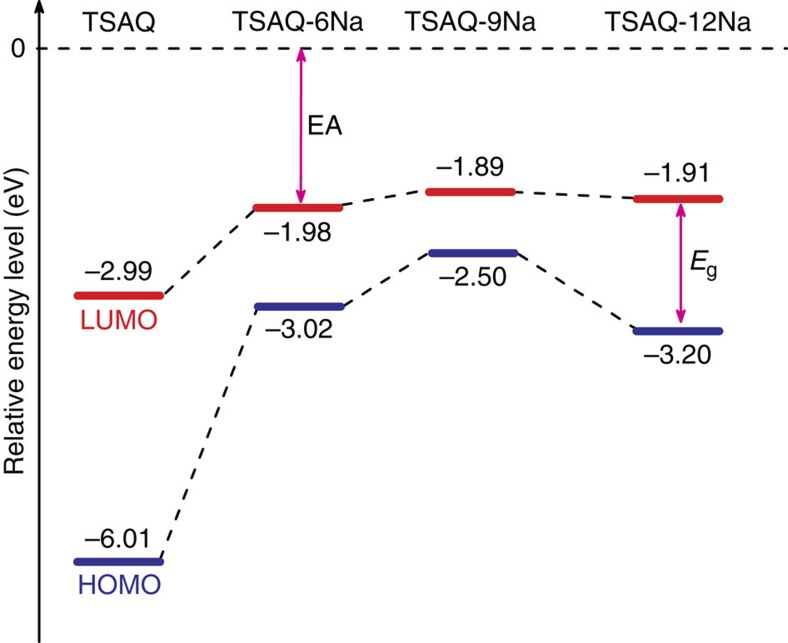
Quantum chemical calculations of Na^+^ storage mechanism. The quantum chemical calculations analysis DFT calculation of the LUMO/HOMO of TSAQ, TSAQ-6Na, TSAQ-9Na and TSAQ-12Na.

The quantum chemical calculations were carried out using GAUSSIAN 09 with the density functional theory (DFT) method with B3LYP exchange-correlation function. According to molecular orbital theory, a lower lowest unoccupied molecular orbital (LUMO) energy means a larger electron affinity and higher reduction potential, while the LUMO–HOMO (highest occupied molecular orbital) gap, usually represented by *E*_g_, is related to the electronic conduction^56^. The electron affinity and *E*_g_ values of the proceeding intermediate are depicted in Fig. 5. The LUMO energy levels of TSAQ-6Na and TSAQ-9Na are pretty close to each other, resulting in a sloping plateau ranging from 0.7 to 0.05 V. The LUMO of TSAQ is 1.01 eV lower than that of TSAQ-6Na, which agrees with a relatively higher plateau centre at about 1.2 V. In addition, DFT calculations (Supplementary Table 1) also show redox reactions of an enolate/quinonoid carbonyl couple (TSAQ/TSAQ-6Na) with an average potential of 1.12 V and a carboxylate/enolate couple (TSAQ-6Na/TSAQ-9Na) with an average potential of 0.74 V, as well as a radical/anion couple (TSAQ-9Na/TSAQ-12Na) with an average potential of 0.36 V. The values agree well with the experimental data^57^. It should be noted that all of the theoretical calculation of the several intermediates show the nearly planar rigid nature, implying a strong sterically hindered effect from the aromatic structure to stabilize the radical intermediates in organic electrodes based on tri-β-ketoamine.

**Figure 6:**
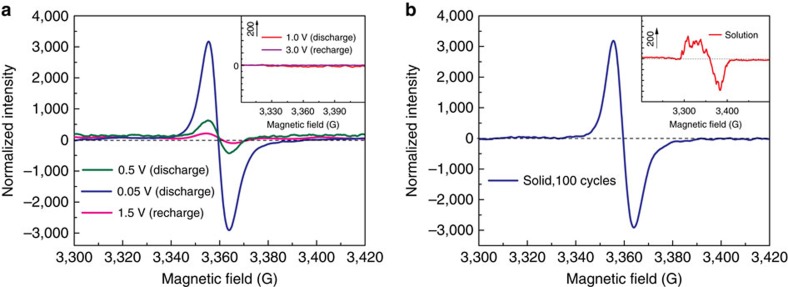
Analysis of the unpaired electron during the electrochemical process. (**a**) CW-EPR for TSAQ at different discharge/charge states. Red (discharge to 1.0 V), olive (discharge to 0.5 V), blue (discharge to 0.05 V), magenta (recharge to 1.5 V) and violet (fully recharge to 3 V); (**b**) Solid-state CW-EPR analysis of TSAQ at 0.05 V (blue) and solution state CW-EPR analysis of the same materials after 100 cycles at 100 mA g^−1^ (insert, red). The electrode was dissolved by toluene for CW-EPR spectrum collection at 190 K.

To investigate the electron transferring and electronic state, electron paramagnetic resonance (EPR) analyses have been carried out on the electrodes with different charge/discharge states. As shown in Fig. 6a, in the sequential EPR profiles, the electrodes at 0.5 and 0.05 V show Gaussian shape peaks centred at 3.370 GHz under the magnetic fields of 9 GHz, while those discharged to 1.0 V and recharged above 1.0 V display no EPR signals. The Landé *g* values turn out to be 1.98, which indicates the spin quantum number of *S*=1/2 or spin multiplicity, verifying the presence of unpaired electron spins. The EPR intensity witnessed an obvious increasing when the electrode was deeply discharged from 0.5 to 0.05 V, implying that the generated unpaired electron became more stable on discharging to a lower voltage. More interestingly, this formation of radical is totally reversible as can be seen from the completely disappearance of the EPR signal after fully recharge and the peak intensity could be almost recovered after discharged to the cutoff voltage of 0.05 V after 100 cycles (Fig. 6b).

To get further insight into the free radical, the electrode discharged to 0.05 V was dissolved in toluene and tested by continuous wave electron paramagnetic resonance (CW-EPR) at 190 K as shown in the inset of Fig. 6b. A hyperfine splitting profile was observed, indicating the profound effects of the surrounding nucleus, particularly that with nuclear spin quantum number *S*≠0 like H (*S*=1/2), Na (*S*=3/2) and N (*S*=1). As a contrast, there are no EPR signals of DCCA detected after 10 cycles at 0.05 V, which again proves a less stable unpaired electron nature of DCCA as compared with TSAA (Supplementary Fig. 15) and TSAQ. According to the singly occupied molecular orbital (SOMO) and spin density distribution (Supplementary Fig. 16) calculated using DFT model, about 80% the unpaired electrons were located on the α-C atom. Generally organic compounds may generate radical species in redox reactions^58^ However, this radical intermediate is usually highly reactive in the electrolyte and rapidly transforms to a dimer, which diminishes the reversibility of the redox process just as the situation for DCAA. EPR analyses unambiguously demonstrate that the extended conjugation as well as rigid structure within TSAA and TSAQ successfully restricts the reactivity of the unpaired electrons of the produced radical intermediates soaking in the electrolyte.

**Figure 7:**
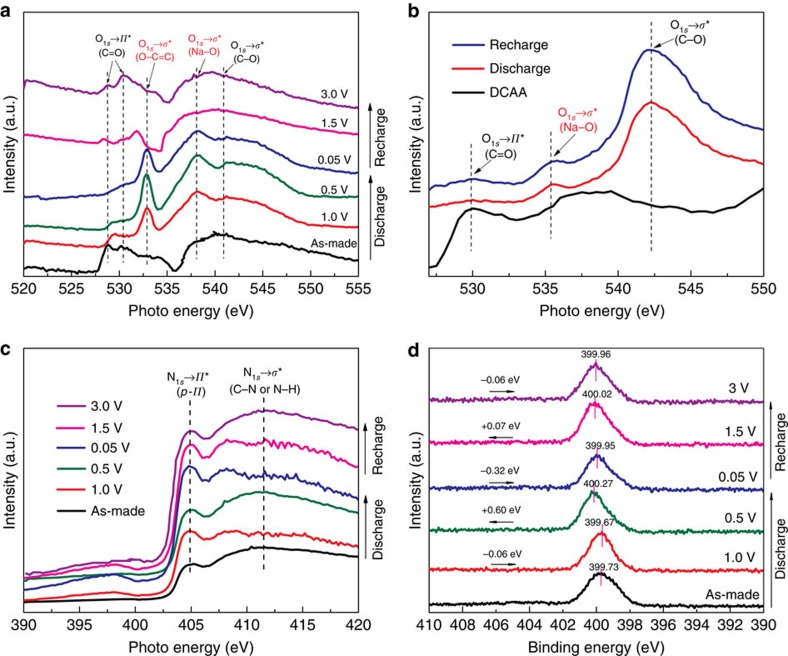
Reversibility analysis of the electrodes. O K-edge XANES spectra of TSAQ (**a**) and DCCA (**b**) electrodes at different states; N K-edge XANES spectra (**c**) and N_1*s*_ XPS (**d**) of the TSAQ electrode at different states. N_1*s*_ spectra, the positive shift of binding energy demonstrates a higher oxidation state than primary state. Comparing with the primary state, the N_1*s*_ variation: 1.0 V (−0.06 eV); 0.5 V (+0.6 eV); 0.05 V (−0.32 eV); 1.5 V (+0.07); and 3 V (−0.06 V). For **a**,**c**,**d**: black, as-made; red, discharge to 1.0 V; olive, discharge to 0.5 V; blue, discharge to 0.05 V; magenta, recharge to 1.5 V; and violet, fully recharge to 3 V.

*Ex situ* Fourier transform infrared spectroscopies (FT-IRs) were conducted to track evolution of functional groups within the electrodes during electrochemical charging and discharging. Unless otherwise specified, 30 wt% super P was substituted by 60 wt% nano-copper powders for the purpose of avoiding extra sodium interaction with the conductive carbon. The O K-edge X-ray absorption near-edge structure (XANES) spectra (Fig. 7a) of the as-prepared TSAQ exhibit sharp peaks at 529 and 530 eV, corresponding to the O_1*s*_→π* (C=O) excitation that originates from the quinone and β-keto, respectively, while the broad absorption in the range of 540–550 eV represents the O_1*s*_→σ* (C–O) excitation^57^ When discharging to 1.0 V, two new strong peaks appear at 533 and 538 eV, attributing to the O_1*s*_→σ* (O–C=C) and O_1*s*_→σ* (O–Na) excitation, respectively. While the peaks at 529 and 530 eV are markedly reduced that means a successful transformation from C=O to C=C–O–Na during sodiation process. After that, the intensity of the peak at 533 eV was gradually enhanced, meanwhile the 529 and 530 eV peaks entirely disappear, implying a complete reduction of C=O. The O K-edge XANES spectra of the electrodes at the fully discharging status show that the C=O in the quinone and β-keto structure has been fully coordinated with Na^+^ before 0.5 V. When the electrodes were recharged back to 3 V, the characteristic peak at 533 eV was markedly weakened and a strong peak at 531.5 eV appeared proving the re-production of C=O bonding inside the structure, suggesting an effective desodiation. The *ex situ* FT-IR spectra shown in Supplementary Fig. 17 also reveal such a highly reversible transformation between C=O and C–O–Na of TSAA and TSAQ electrode^25^. The above infrared and O K-edge XANES analysis of the electrodes under different discharge/charge states unambiguously confirm the highly reversible procedure of the Na^+^ insertion/extraction. These results clearly verify the excellent reversibility of the tri-β-ketoenamine-linked compounds. The O K-edge XANES spectra of DCCA were shown in Fig. 7b. The peak at 530 eV represents the O_1*s*_→π* (C=O) excitation, which completely disappeared when discharged to 0.05 V. However, when the voltage was increased to 3.0 V, the characteristic excitation of C=O group hardly recovered. Moreover, the peaks that were ascribed to the O_1*s*_→σ* (C–O) and O_1*s*_→σ* (O–Na) excitations display little variation between the discharged and recharged states. Such an irreversible transformation from C=O and C–O–Na groups results in a poor cyclability of DCCA electrode as expected. After fully recharging to 3 V, the peak at ≈1,674 cm^−1^ emerges again, indicating well recuperation of the carbonyl groups after the desodiation process. Therefore, the naturally robust structure and sufficient conjugate action from aromatic amine have effectively suppressed the side reaction of the α-C radical, leading to considerable enhancement of the reversibility between C=O and C–O–Na transformation, thus stable cycling performance has been achieved^59^.

The aforementioned results and discussions clearly demonstrate that there are obvious *p*-*π* resonance effect between the non-bonding electrons of aromatic amine (>N–) and the connecting tri-β-keto in the TSAQ electrode. The N K-edge XANES spectra (Fig. 7c) and X-ray photoelectron spectroscopy (XPS) spectra (Fig. 7d and Supplementary Fig. 18) were used to verify the specific transformation of the electrodes at different stages of charge and discharge. The binding energies were referenced to C_1*s*_ line at 284.8 eV (±0.1 eV) from graphite carbon (Supplementary Fig. 19). All of the spectra exhibited a characteristic peak at 405 eV, corresponding to the N_1*s*_→π* excitation, which results from the energy transition from the N_1*s*_ to the π* of the aromatic ring and the ketene (N–C=C). The broad absorption above 406 eV represents N_1*s*_→σ* (C–N or N–H) excitation^60^. For the electrode discharged to 1.0 V, the peak intensity at 405 eV steadily increases, demonstrating an enhanced *p*-*π* Hückel effect of the generated anthranol from the reduction of anthraquinone. However, as the proceeding of the discharge of the electrode to a voltage below 0.5 V, the signal due to N_1*s*_→π* excitation decreased. This could be explained by the formation of electrophilic α-C radical intermediates, which causes a higher oxidation state of neighbouring N atoms thus leading to a remarkably increasing of the N_1*s*_ binding energy (+0.60 eV). After that, the N_1*s*_ binding energy witnesses a decline from 400.27 at 0.5 V to 399.95 eV at 0.05 V, an indication of receiving electrons and coordinating with Na^+^. Furthermore, the N_1*s*_→π* excitation reaches the maximum intensity at 0.05 V that is characteristic of a *sp*^2^ structure for α-C anion, revealing a weak interaction between anion and Na^+^. After fully recharged to 3 V, the intensity of N_1*s*_→π* excitation recovers to the initial value, which implies a strong reversible evolution of *p*-*π* delocalization. The results of N K-edge XANES and XPS spectra of the TSAQ electrode at different discharge states indicate a remarkable electron resonance effect of the amine (>N–) to stabilize the α-C radical intermediates. The variations of intensity of such an effect verify the completely reversible transformation within the three states of the α-C: C=C; –C· radical; and –C^−^ anion. The ^13^C NMR spectra shown in Supplementary Fig. 20 indicate a higher chemical shift of about 161.2 p.p.m. for the α-C discharged to 0.05 V as compared with the pristine electrode (146.8 p.p.m.). Such chemical shifts illustrate an increased electron resonance effect and shielding effect.

**Figure 8:**
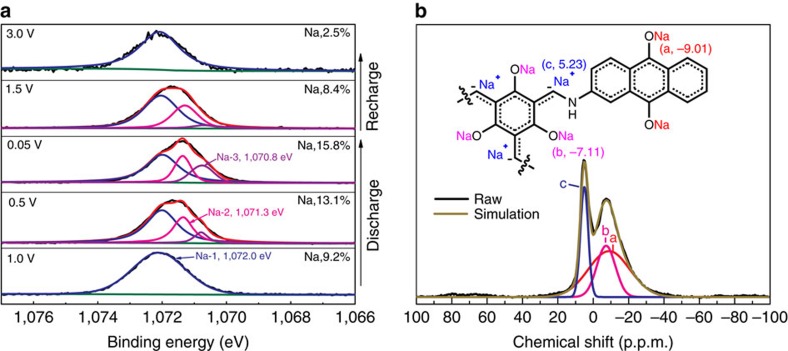
Analysis of sodium insertion to α-C radical. (**a**) Na_1*s*_ XPS spectra of the TSAQ electrode under different states. The sodium atom content is 9.2%, 13.1%, 15.8%, 8.4% and 2.5% at the state of discharge to 1.0 V, discharge to 0.5 V, discharge to 0.05 V, recharge to 1.5 V and fully recharge to 3 V, respectively. Sodium ratio (Na-1:Na-2:Na-3): 1.0 V (≈1:0:0); 0.5 V (2:1:0.3); 0.05 V (2:1:1); and 1.5 V (2:1:0.1). Black (raw), red (simulation), olive (background), blue (1,072.0 eV, Na-1), magenta (1,071.3 eV, Na-2) and violet (1,070.8 eV, Na-3). (**b**) Solid-state ^23^Na nuclear magnetic resonance (NMR) spectrum of the TSAQ electrode after discharged to 0.05 V. Black (raw), dark yellow (simulation), red (a, −9.01 p.p.m.), magenta (b, −7.11 p.p.m.) and olive (c, 5.23 p.p.m.).

The XPS spectra of the Na_1*s*_ signal (Fig. 8a) of the TSAQ electrode under different charge/discharge states could be deconvoluted into three peaks, which indicate that the sodium ions were inserted into the TSAQ electrode at three different sites. The peak at 1,072.0 eV could be ascribed to the sodium ions in O–Na groups originating from reduction of quinone structure (Na-1). While the peak for O–Na groups due to the reduction of β-keto (Na-2) was located at 1,071.3 eV. The peak at 1,070.8 eV represents the Na^+^ intercalated into the carbon (C–Na, Na-3). The Na-3 signal emerged at around 0.5 V (Na ration, Na-1:Na-2:Na-3≈2:1:0.3), and then reached the maximum value at 0.05 V (Na ration, Na-1:Na-2:Na-3≈2:1:1), after recharging back to 1.5 V, when the sodiation was almost completed (Na ration, Na-1:Na-2:Na-3≈2:1:0.1). Considering the above results, the α-C^−^ anion contributes quarter of the total sodium storage capacity for TSAQ, which is in good agreement with the aforementioned electrochemical reaction mechanism. Solid-state ^23^Na NMR analysis (Fig. 8b) has been performed on the sample in the fully discharged state. The raw NMR data could be perfectly deconvoluted into three kinds of signals (*a*, *b* and *c*), which are in good agreement with the Na_1*s*_ XPS results. The proportional to the integral area could be defined as the percentage of sodium content of these three well-deconvoluted peaks. According to the literatures^61^, broad lines are ascribed to cations with restricted mobility and narrower lines represent mobile cations. Thus, the broad peak ranging between 0 and −40 p.p.m. indicates sodium signal with more restricted mobility, while the narrow peaks centred at 5.23 p.p.m. are representative of sodium inserted to carbon frameworks with ionic character. Therefore, the signal at 5.23 p.p.m. could be attributed to the sodium inserted into the unsaturated carbon with more ionic character due to the weak interaction with carbon atoms, which is corresponding to the Na-3 signal of the XPS. The *ex situ* Raman spectra (Supplementary Fig. 21) of the TSAQ electrode also display a reversible peak centred at about 360 cm^−1^, which also reveals the strong Na/C interaction of the anion after sodiation. The broad signal at −7.11 and −9.01 p.p.m. in the NMR spectrum could be ascribed to the Na-2 and Na-3 signal, which represents sodium insertion with restricted mobility at the carbonyl groups. The percentage of sodium inserted into carbon, and the sodium coordinated with C=O groups of β-keto and quinone are about 25%, 25% and 50% according to the integral peak area from the simulated profile, respectively. These results again confirm the α-C radical intermediate contributes nearly 25% of the total capacity. Such a value could also be certified by the energy-dispersive spectroscopy (EDS) elemental mapping using TEM methods (Supplementary Fig. 22) we could see the homogeneous distribution of nitrogen, sodium, carbon and oxygen element. Also after fully discharged to 0.05 V, the EDS data of Na/O atomic ration is 1.19, which is very close to the estimated theoretical value of 1.33 by the molecular TSAQ-12Na. As a result, the tri-β-ketoenamine structure contributes remarkable Na/C interaction via α-C radical. On the larger size of sodium ion radius and naturally weaker C–Na bond than the counterparts of lithium ions, rare works refer to effective sodium-ion intercalation into the unsaturated frameworks (hard carbon (Na/C_17_) and graphite (Na/C_64_))^62^,^63^,^64^, resulting in an unpractical negative capacity^3^,^65^,^66^,^67^. In this work, the generated α-C radical shows obvious and reversible Na^+^ insertion (more than 0.83 Na^+^ per radical), which provides the electrode with exceptional stable and high capacity. The electrochemical behaviour is very similar to the pristine *n*-type radical polymers having reversible electrochemical reaction between a radical and an anion^36^.

In summary, we report a strategy to design highly durable organic electrodes for sodium-ion batteries. Through a resonance effect of the *p*-*π* conjunction and the generated 9-*π*-electron aromatic Hückel ring, as well as the steric effect of the rigid geometry, we have successfully developed a stable α-C radical intermediate, which effectively prevents the formation of inactive dimer or other materials, thereby ensuring excellent cycling stability. The tri-carbonyl-based organic electrodes, TSAA and TSAQ, show excellent reversible electro-transformation between C=O and C–O–Na, and deliver no obvious capacity degradation after more than 2,500 cycles at a high current density of 1 A g^−1^. We also found that the α-C radical intermediate displays weak electron affinity and thereby resulting in α-C anion at a low potential (<0.5 V, versus Na/Na^+^), which facilitates the ‘bonding' behaviour between Na^+^ and carbon atoms. As a result, the electrodes contribute to strong Na/C interaction, the overall amount of Na^+^ insertion into carbon atoms is close to one ion per C radical. The strategy of intermediate radical stabilization to enhance cycling life, as well as energy storage capability could benefit the design of next-generation high-performance organic electrode materials for NIBs, and this idea may also be extended to design organic electrodes for other energy storage systems.

## Methods

### Synthesis of TSAA and TSAQ

A 50 ml three-necked bottle was charged with 2-Anthramine/2-Anthraquinoylamine (1.42 mmol) and 1,3,5-triformylphloroglucinol (0.96 mmol). Then the reaction solvent *N*, *N*-dimethylformamide (DMF) (20 ml) was added and the resulting suspension was heated to 120 °C and kept for 48 h. The resulting solids were isolated by filtration and washed with acetone for three times (20 ml × 3) and then ethanol for another three times (20 ml × 3), followed by a 6 h Soxhlet extraction using HCN as eluting solvent, and finally dried under high vacuum at 80 °C. TSAA was obtained as an orange powder while TSAQ was got as brown powder (both yield >90%). For the poor solubility in common solvents like dimethylsulphoxide, CHCl_3_ and acetone, the exactly solvent state ^1^H NMR data were hard obtained. Solid-state ^13^C NMR, FT-IR, high-resolution mass spectrometry (HRMS), as well as element analysis methods were used to confirm the structures.

### Structure characterization

^1^H NMR (500 MHz) spectra were obtained with a Bruker spectrometer at an operating temperature of 25 °C using D_2_SO_4_ as solvent. Solid-state CP/MAS ^13^C/^23^Na NMR (100 MHz) spectra were obtained with a Bruker spectrometer at an operating temperature of 25 °C at a spin rate of 10 kHz. Mass spectrometry analysis was performed with a Thermo Q Exactive mass spectrometer (Thermo Fisher Scientific, USA). The mass spectrometer was operated in positive mode with a spray voltage of 3.5 KV, capillary temperature of 320 °C, sheath gas flow of 30, auxiliary gas of 10 (a.u.). CHN analysis was performed on a Vario ELIII CHNOS Elementar analysator from Elementar Analysensysteme GmbH. FT-IR spectra were collected with a VARIAN 1000 FT-IR (scimitar series) spectrometer in the 400–4,000 per cm region (KBr pressed disks).

### Tris *N*-salicylideneanthramine

^1^H NMR (500 MHz, D_2_SO_4_): *δ*10.05 (s, Ar–NH); 9.59 (s, Ar–NH); 9.48 (d, Ar–NH) (these four resonances are due to 3H); 9.60–9.48 (s, 6H, Ar-H); 8.81–8.75 (s, 3H, =C–H); 9.16–8.84 (m, 9H); and 8.75–8.22 (m, 12H). ^13^C NMR (100 MHz, solid): *δ*185.56; 145.85; 136.36; 131.62; 129.96; 126.59; 116.28; and 107.92. IR (solid, ATR): 3,033; 2,956; 1,601; 1,593; 1,548; 1,437; 1,354; 1,254; 1,073; 1,006; 895; 810; and 729 cm^−1^. HRMS (*m*/*z*): [M+H]^+^ calcd. for C_51_H_34_N_3_O_3_^+^, 736.2594; found, 736.2625; analysis (calcd. found for C_51_H_33_N_3_O_3_): C (83.25, 82.88); H (4.52, 4.32); and N (5.71, 5.57).

### TSAQ

^1^H NMR (500 MHz, D_2_SO_4_): δ10.22 (d, Ar–NH); 10.09 (s, Ar–NH); 9.62 (d, Ar–NH) (these four resonances are due to 3H); 8.75–8.80 (m, 3H); 8.82–8.70 (m, 3H, =C–H); 8.70–8.52 (m, 9H, Ar–H); and 8.32–8.49 (m, 12H, Ar–H). ^13^C NMR (100 MHz, solid): 185.21; 181.04; 147.80; 143.35; 134.71; 131.92; 129.13; 118.52; and 109.04. IR (solid, ATR): 3,355; 3,048; 1,671; 1,622; 1,568; 1,455; 1,323; 1,269; 1,089; 928; 845; and 713 cm^−1^. HRMS (*m*/*z*): [M+H]^+^ calcd. for C_51_H_28_N_3_O_9_^+^, 826.1820; found, 826.1802; analysis (calcd., found for C_51_H_27_N_3_O_9_): C (74.18, 73.84); H (3.3, 3.22); and N (5.09, 4.87).

### Morphological and thermal stability analysis

Powder X-ray diffraction data were collected over the 2*θ* range 2°−40° on a CPS120 Inel diffractometer equipped with Ni-filtered Cu Kα radiation (40 kV, 100 mA) at room temperature with a scan speed of 2° min^−1^. High-resolution transmission electron microscopy images were collected in a JEOL JEM-2100F microscope at an accelerating voltage of 200 kV. SEM experiments were performed on a FEI Quanta-200 scanning electron microscope. TGA was performed at a heating rate of 10 K min^−1^ under Ar atmosphere using a PerkinElmer TGA 7.

### *Ex situ* analysis

XANES experiments were performed at the Soft X-ray Spectroscopy station and Photoelectron Spectroscopy station. The electron beam energy of the storage ring was 3.5 GeV with a maximum stored current of 300 mA. The spectra were normalized to the incident photon flux. XPS was performed on a ESCALAB 250 photoelectron spectrometer (ThermoFisher Scientific) with Al Ka (1486.6 eV) as the X-ray source set at 150 W and a pass energy of 30 eV for high-resolution scan, the base pressure was 3 × 10^−9^ mbar, and the binding energies were referenced to C1s line at 284.8 eV from graphite carbon. Before the test, the charged/discharged electrodes were disassembled from coin-type cells in an argon-filled glove box, washed with EC several times, and dried under vacuum at 60 °C for 24 h. The continuous wave electron paramagnetic resonance (CW-EPR) spectra of the compounds were recorded with a Bruker ER200-SRC-10/12 X band spectrometer with an amplitude modulation of 0.8 G and microwave power of 1 mW. The Raman spectra of samples were obtained on a Lab RAM HR 800 Raman microscope with an excitation laser beam wavelength of 633 nm. Before the test, the charged/discharged electrodes were disassembled from coin-type cells in an argon-filled glove box, washed with DMC several times and dried under vacuum.

### Electrochemical characterization

Samples of electrochemically active materials were mixed with active material, super P and polyvinylidene fluoride in a 60:30:10 weight ratio. After ball-mixing with N-methyl-2-pyrrolidone (NMP) for 4 h, the mixture was coated uniformly using a doctor-blade on the copper foil with roughly 0.7 mg cm^−2^ mass loading. The electrodes were dried at 110 °C under vacuum for overnight. The electrochemical performance was evaluated using 2016 coin cells with a sodium metal anode and NaClO_4_ (1 M) in EC/DMC (1:1 w/w) as the electrolyte. The samples for *ex situ* experiment such as FT-IR, X-ray absorption near-edge structure (XNEFS), XPS, solid state NMR, TEM and EDS were prepared by replacing acetylene black with equal mass Nano-copper. The CV measurements were carried out using CHI660 electrochemical workstation. Electrochemical impedance spectroscopy was recorded on Solartron 1470E, the amplitude of the sine perturbation signal was 5 mV and the frequency was scanned from the highest (10 kHz) to the lowest (5 mHz). Galvanostatic charge discharge cycles were tested by Neware battery tester at different current densities at room temperature.

### Data availability

The data that support the findings of this study are available from the corresponding author on request.

## Additional information

**How to cite this article:** Wu, S. *et al*. Highly durable organic electrode for sodium-ion batteries via a stabilized α-C radical intermediate. *Nat. Commun.* **7,** 13318 doi: 10.1038/ncomms13318 (2016).

**Publisher's note:** Springer Nature remains neutral with regard to jurisdictional claims in published maps and institutional affiliations.

## Supplementary Material

Supplementary Information Supplementary Figures 1- 22, Supplementary Table 1, Supplementary Note 1 and Supplementary References

## Figures and Tables

**Figure 1:**
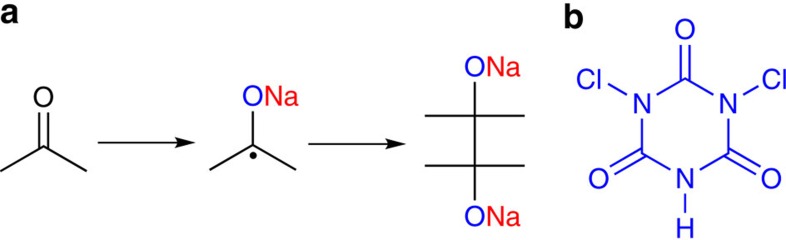
Reactivity of radical intermediates. (**a**) Dimerization of a simple carbonyl radical intermediate. (**b**) Dichloroisocyanuric acid.

**Figure 2:**
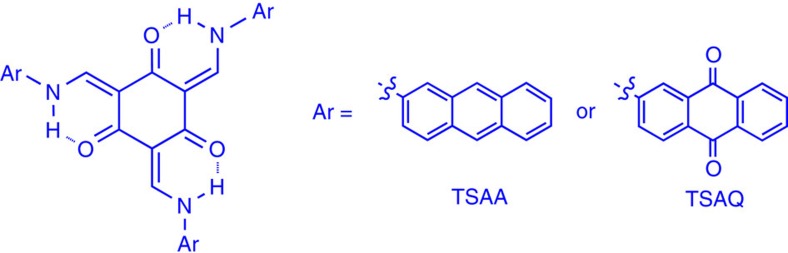
Design of β-ketoenamine-linked compound-based organic electrode. Chemical structure of TSAA and TSAQ.

## References

[b1] Palomares V., Casas-Cabanas M., Castillo-Martinez E., Han M. H. & Rojo T. Update on Na-based battery materials. A growing research path. Energy Environ. Sci. 6, 2312–2337 (2013).

[b2] Park Y. U. . A new high-energy cathode for a Na-ion battery with ultrahigh stability. J. Am. Chem. Soc. 135, 13870–13878 (2013). 23952799 10.1021/ja406016j

[b3] Masse R. C., Uchaker E. & Cao G. Z. Beyond Li-ion: electrode materials for sodium- and magnesium-ion batteries. Sci. China Mater. 58, 715–766 (2015).

[b4] Lu X. . Liquid-metal electrode to enable ultra-low temperature sodium-beta alumina batteries for renewable energy storage. Nat. Commun. 5, 4758 (2014). 25081362 10.1038/ncomms5578

[b5] Li Y., Hu Y. S., Li H., Chen L. & Huang X. A superior low-cost amorphous carbon anode made from pitch and lignin for sodium-ion batteries. J. Mater. Chem. A 4, 96–104 (2016).

[b6] Sun Y. . Direct atomic-scale confirmation of three-phase storage mechanism in Li_4_Ti_5_O_12_ anodes for room-temperature sodium-ion batteries. Nat. Commun. 4, 1870 (2013). 23695664 10.1038/ncomms2878

[b7] Wang Y. . Ti-substituted tunnel-type Na_0. 44_MnO_2_ oxide as a negative electrode for aqueous sodium-ion batteries. Nat. Commun. 6, 6401 (2015). 25806965 10.1038/ncomms7401

[b8] Wen Y. . Expanded graphite as superior anode for sodium-ion batteries. Nat. Commun. 5, 4033 (2014). 24893716 10.1038/ncomms5033

[b9] Wenzel S., Hara T., Janek J. & Adelhelm P. Room-temperature sodium-ion batteries: improving the rate capability of carbon anode materials by templating strategies. Energ. Environ. Sci. 4, 3342–3345 (2011).

[b10] Xu J. . High-performance sodium ion batteries based on a 3D anode from nitrogen-doped graphene foams. Adv. Mater. 27, 2042–2048 (2015). 25689053 10.1002/adma.201405370

[b11] Zheng F., Yang Y. & Chen Q. High lithium anodic performance of highly nitrogen-doped porous carbon prepared from a metal-organic framework. Nat. Commun. 5, 5261 (2014). 25374050 10.1038/ncomms6261

[b12] Su D., Dou S. & Wang G. Bismuth: a new anode for the Na-ion battery. Nano Energy 12, 88–95 (2015).

[b13] Xiao L. . High capacity, reversible alloying reactions in SnSb/C nanocomposites for Na-ion battery applications. Chem. Commun. 48, 3321–3323 (2012). 10.1039/c2cc17129e 22361490

[b14] Darwiche A. . Better cycling performances of bulk Sb in Na-ion batteries compared to Li-ion systems: an unexpected electrochemical mechanism. J. Am. Chem. Soc. 134, 20805–20811 (2012). 23194439 10.1021/ja310347x

[b15] Xu Y., Zhu Y., Liu Y. & Wang C. Electrochemical performance of porous carbon/tin composite anodes for sodium-ion and lithium-ion batteries. Adv. Energy Mater. 3, 128–133 (2013).

[b16] Stevens D. A. & Dahn J. R. The mechanisms of lithium and sodium insertion in carbon materials. J. Electrochem. Soc. 148, A803–A811 (2001).

[b17] Wang Y., Xiao R., Hu Y. S., Avdeev M. & Chen L. P2-Na_0.6_[Cr_0.6_Ti_0.4_]O_2_ cation-disordered electrode for high-rate symmetric rechargeable sodium-ion batteries. Nat. Commun. 6, 6954 (2015). 25907679 10.1038/ncomms7954 PMC4421853

[b18] Sun J. . A phosphorene-graphene hybrid material as a high-capacity anode for sodium-ion batteries. Nat. Nanotechnol. 10, 980–985 (2015). 26344183 10.1038/nnano.2015.194

[b19] Wang C. . Extended pi-conjugated system for fast-charge and discharge sodium-ion batteries. J. Am. Chem. Soc. 137, 3124–3130 (2015). 25665063 10.1021/jacs.5b00336

[b20] Sakaushi K. . Aromatic porous-honeycomb electrodes for a sodium-organic energy storage device. Nat. Commun. 4, 1485 (2013). 23403585 10.1038/ncomms2481

[b21] Chen H. . Lithium salt of tetrahydroxybenzoquinone: toward the development of a sustainable Li-ion battery. J. Am. Chem. Soc. 131, 8984–8988 (2009). 19476355 10.1021/ja9024897

[b22] Wang S. . All organic sodium-ion batteries with Na_4_C_8_H_2_O_6_. Angew. Chem. Int. Ed. 53, 5892–5896 (2014). 10.1002/anie.201400032 24677513

[b23] Song Z., Zhan H. & Zhou Y. Polyimides: promising energy-storage materials. Angew. Chem. Int. Ed. 49, 8444–8448 (2010). 10.1002/anie.201002439 20862664

[b24] Castillo-Martínez E., Carretero-González J. & Armand M. Polymeric schiff bases as low-voltage redox centers for sodium-ion batteries. Angew. Chem. Int. Ed. 53, 5341–5345 (2014). 10.1002/anie.201402402 24757125

[b25] Wu X. . Unraveling the storage mechanism in organic carbonyl electrodes for sodium-ion batteries. Sci. Adv. 1, e1500330 (2015). 26601260 10.1126/sciadv.1500330 PMC4643786

[b26] Haupler B., Wild A. & Schubert U. S. Carbonyls: powerful organic materials for secondary batteries. Adv. Energy Mater. 5, 1402034 (2015).

[b27] Wu X. . A spray drying approach for the synthesis of a Na_2_C_6_H_2_O_4_/CNT nanocomposite anode for sodium-ion batteries. J. Mater. Chem. A 3, 13193–13197 (2015).

[b28] Nokami T. . Polymer-bound pyrene-4, 5, 9, 10-tetraone for fast-charge and -discharge lithium-ion batteries with high capacity. J. Am. Chem. Soc. 134, 19694–19700 (2012). 23130634 10.1021/ja306663g

[b29] Tobishima S., Yamaki J. & Yamaji A. Cathode characteristics of organic electron-acceptors for lithium batteries. J. Electrochem. Soc. 131, 57–63 (1984).

[b30] Pasquali M., Pistoia G., Boschi T. & Tagliatesta P. Redox mechanism and cycling behavior of nonylbenzo-hexaquinone electrodes in Li Cells. Solid State Ion 23, 261–266 (1987).

[b31] Williams D. L., Byrne J. J. & Driscoll J. S. A high energy density lithium/dichloroisocyanuric acid battery system. J. Electrochem. Soc. 116, 2–4 (1969).

[b32] Wang H. G. . Tailored aromatic carbonyl derivative polyimides for high-power and long-cycle sodium-organic batteries. Adv. Energy Mater. 4, 403–410 (2014).

[b33] Zhu L. M. . Self-doped polypyrrole with ionizable sodium sulfonate as renewable cathode material for sodium ion batteries. Chem. Commun. 49, 11370–11372 (2013). 10.1039/c3cc46642f 24162858

[b34] Nesvadba P., Bugnon L., Maire P. & Novak P. Synthesis of a novel spirobisnitroxide polymer and its evaluation in an organic radical battery. Chem. Mater. 22, 783–788 (2010).

[b35] Bergner B. J., Schurmann A., Peppler K., Garsuch A. & Janek J. TEMPO: a mobile catalyst for rechargeable Li-O_2_ batteries. J. Am. Chem. Soc. 136, 15054–15064 (2014). 25255228 10.1021/ja508400m

[b36] Suga T., Ohshiro H., Sugita S., Oyaizu K. & Nishide H. Emerging N-type redox-active radical polymer for a totally organic polymer-based rechargeable battery. Adv. Mater. 21, 1627–1630 (2009). 10.1002/adma.201003525 21287636

[b37] Liang Y., Tao Z. & Chen J. Organic electrode materials for rechargeable lithium batteries. Adv. Energy Mater. 2, 742–769 (2012).

[b38] Oyaizu K., Ando Y., Konishi H. & Nishide H. Nernstian adsorbate-like bulk layer of organic radical polymers for high-density charge storage purposes. J. Am. Chem. Soc. 130, 14459–14461 (2008). 18842045 10.1021/ja803742b

[b39] Guo W., Yin Y. X., Xin S., Guo Y. G. & Wan L. J. Superior radical polymer cathode material with a two-electron process redox reaction promoted by graphene. Energy Environ. Sci. 5, 5221–5225 (2012).

[b40] Imada Y. . Isolation of hypervalent group-16 radicals and their application in organic-radical batteries. J. Am. Chem. Soc. 138, 479–482 (2015). 10.1021/jacs.5b10774 26721786

[b41] Zeng Z. . Pro-aromatic and anti-aromatic π-conjugated molecules: an irresistible wish to be diradicals. Chem. Soc. Rev. 44, 6578–6596 (2015). 25994857 10.1039/c5cs00051c

[b42] Back O., Donnadieu B., Parameswaran P., Frenking G. & Bertrand G. Isolation of crystalline carbene-stabilized P-2-radical cations and P-2-dications. Nat. Chem. 2, 369–373 (2010). 20414236 10.1038/nchem.617

[b43] Zeng W. . Super-heptazethrene. Angew. Chem. Int. Ed. 55, 8615–8619 (2016). 10.1002/anie.201602997 27240255

[b44] Ulas G., Lemmin T., Wu Y., Gassner G. T. & DeGrado W. F. Designed metalloprotein stabilizes a semiquinone radical. Nat. Chem. 8, 354–359 (2016). 27001731 10.1038/nchem.2453 PMC4857601

[b45] Perkins C. W., Clarkson R. B. & Martin J. C. A persistent T-shaped 9-S-3 pi-sulfuranyl radical-electron-spin-resonance and electron-spin echo studies. J. Am. Chem. Soc. 108, 3206–3210 (1986).

[b46] Troy T. P. . Hydroxyl addition to aromatic alkenes: resonance-stabilized radical intermediates. J. Phys. Chem. A 116, 7906–7915 (2012). 22770493 10.1021/jp304875r

[b47] Yelamaggad C. V., Achalkumar A. S., Rao D. S. S. & Prasad S. K. A new class of discotic mesogens derived from Tris(N-salicylideneaniline)s existing in C-3 h and C-s keto-enamine forms. J. Org. Chem. 72, 8308–8318 (2007). 17915924 10.1021/jo0712650

[b48] DeBlase C. R., Silberstein K. E., Truong T. T., Abruna H. D. & Dichtel W. R. Beta-ketoenamine-linked covalent organic frameworks capable of pseudocapacitive energy storage. J. Am. Chem. Soc. 135, 16821–16824 (2013). 24147596 10.1021/ja409421d

[b49] Patra B. C., Khilari S., Satyanarayana L., Pradhan D. & Bhaumik A. A new benzimidazole based covalent organic polymer having high energy storage capacity. Chem. Commun. 52, 7592–7595 (2016). 10.1039/c6cc02011a 27222226

[b50] Renault S. . Superlithiation of organic electrode materials: the case of dilithium benzenedipropiolate. Chem. Mater. 28, 1920–1926 (2016).

[b51] DeBlase C. R. . Cation-dependent stabilization of electrogenerated naphthalene diimide dianions in porous polymer thin films and their application to electrical energy storage. Angew. Chem. Int. Ed. 54, 13225–13229 (2015). 10.1002/anie.201505289 26355871

[b52] Chandra S. . Chemically stable multilayered covalent organic nanosheets from covalent organic frameworks via mechanical delamination. J. Am. Chem. Soc. 135, 17853–17861 (2013). 24168521 10.1021/ja408121p

[b53] Chong J. H., Sauer M., Patrick B. O. & MacLachlan M. J. Highly stable keto-enamine salicylideneanilines. Org. Lett. 5, 3823–3826 (2003). 14535719 10.1021/ol0352714

[b54] Zhang N. . Spherical nano-Sb@C composite as high-rate and ultra-stable anode material for sodium-ion batteries. Nano Res. 8, 3384–3393 (2015).

[b55] Liu H. . Nitrogen-doped carbon/graphene hybrid anode material for sodium-ion batteries with excellent rate capability. J. Power Sources 319, 195–201 (2016).

[b56] Song Z. . A quinone-based oligomeric lithium salt for superior Li-organic batteries. Energy Environ. Sci. 7, 4077–4086 (2014).

[b57] Wang H. . Renewable-juglone-based high-performance sodium-ion batteries. Adv. Mater. 27, 2348–2354 (2015). 25728939 10.1002/adma.201405904

[b58] Armand M. . Conjugated dicarboxylate anodes for Li-ion batteries. Nat. Mater. 8, 120–125 (2009). 19151701 10.1038/nmat2372

[b59] Yabuuchi N., Kubota K., Dahbi M. & Komaba S. Research development on sodium-ion batteries. Chem. Rev. 114, 11636–11682 (2014). 25390643 10.1021/cr500192f

[b60] Shimada H. . Nitrogen K-edge X-ray absorption near edge structure of pyrimidine-containing nucleotides in aqueous solution. J. Chem. Phys. 142, 175102 (2015). 25956126 10.1063/1.4919744

[b61] Alcantara R., Lavela P., Ortiz G. F. & Tirado J. L. Carbon microspheres obtained from resorcinol-formaldehyde as high-capacity electrodes for sodium-ion batteries. Electrochem. Solid-State Lett. 8, A222–A225 (2005).

[b62] Ge P. & Fouletier M. Electrochemical intercalation of sodium in graphite. Solid State Ion 28, 1172–1175 (1988).

[b63] Asher R. & Wilson S. Lamellar compound of sodium with graphite. Nature 181, 409–410 (1958).

[b64] Han X., Qing G., Sun J. & Sun T. How many lithium ions can be inserted onto fused C6 aromatic ring systems? Angew. Chem. Int. Ed. 51, 5147–5151 (2012). 10.1002/anie.201109187 22511505

[b65] DiVincenzo D. & Mele E. Cohesion and structure in stage-1 graphite intercalation compounds. Phys. Rev. B 32, 2538 (1985). 10.1103/physrevb.32.2538 9937330

[b66] Sangster J. C-Na (carbon-sodium) system. J. Phase Equilib. Diff. 28, 571–579 (2007).

[b67] Luo W., Allen M., Raju V. & Ji X. An organic pigment as a high-performance cathode for sodium-ion batteries. Adv. Energy Mater. 4, 1400554 (2014).

