# Supplementary material for: Highly durable organic electrode for sodium-ion batteries via a stabilized α-C radical intermediate
Source: Nat Commun. 2016 Nov 7;7:13318. doi: 10.1038/ncomms13318 (PMC5103065; doi:10.1038/ncomms13318)
Supplement: Supplementary Information — Supplementary Figures 1- 22, Supplementary Table 1, Supplementary Note 1 and Supplementary References [file ncomms13318-s1.pdf]

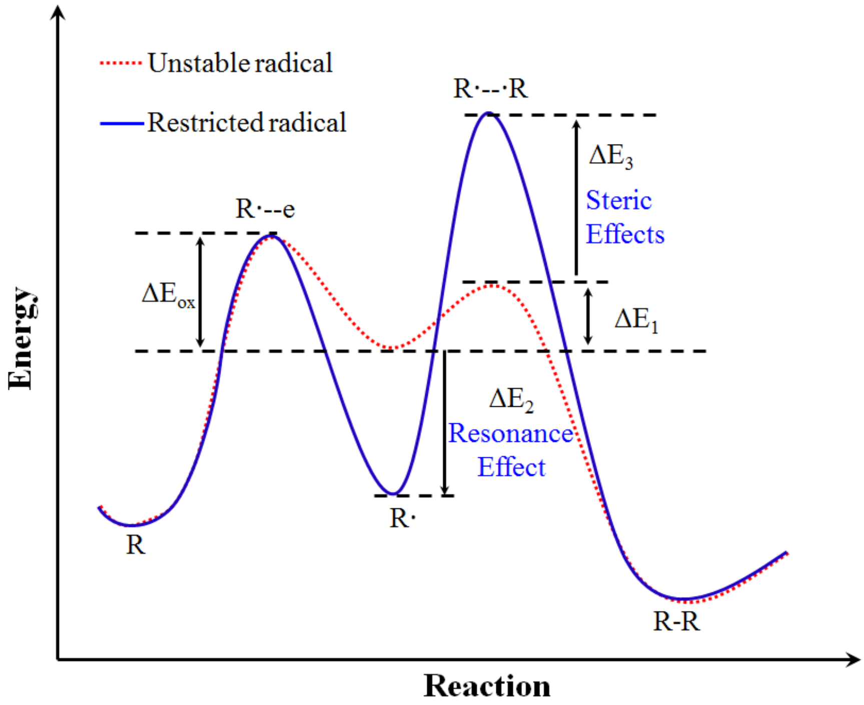

Supplementary Figure 1: The kinetic investigation of the radicals during electrochemical process. R (electrode materials), R·--e (received one electron), R· (radical intermediate), R·--·R (dimer transition state) and R-R (dimer). ΔE refers to the reaction barrier.

The reaction from R· to R-R has a transition state R·--·R, and the energy level could be represented as the red line. The reaction forming a dimer is quite easy because of the low energy gap, ΔE_1. As depicted in the following green line, the electron resonance effect stabilizes the radical compound R· by reducing the energy level about ΔE_2, while the hinder effect restricts the radical reactivity by improving the energy level (represented by ΔE_3) of R·--·R. As a result of a higher energy gap (ΔE_1+ΔE_2+ΔE_3) between the restricted radical and its transition state than that of the instable radical, thus the combination rate of the inactive dimer (R-R) could be geometrically decreased.

All reagents were obtained from commercial suppliers and used as received unless otherwise noted. All air-sensitive manipulations were carried out under nitrogen atmosphere by standard Schlenk-line techniques. 1,3,5-triformylphloroglucinol (TFP) was prepared and characterized according to literature[2].

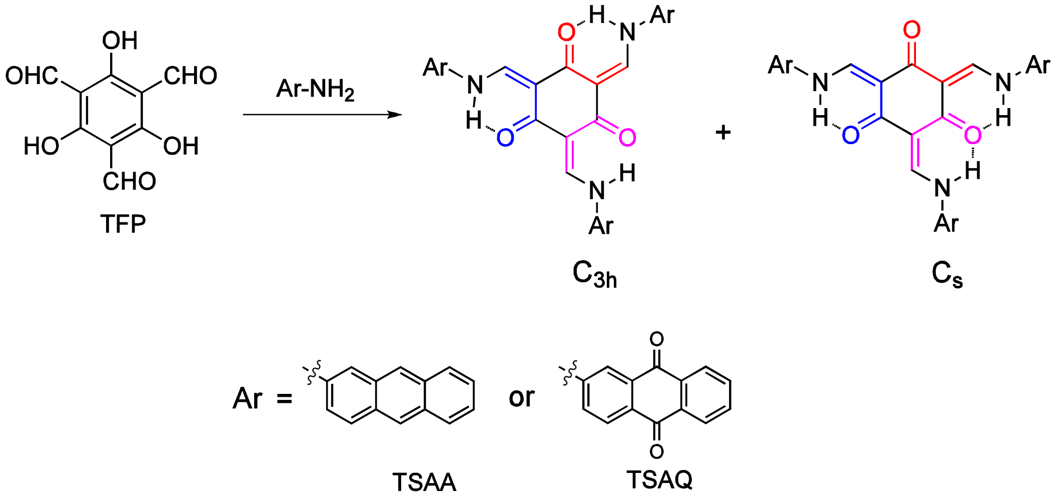

Supplementary Figure 2: Synthetic routine for TSAA and TSAQ. Both of them show two isomers.The synthetic method for Tris(N-salicylidene anthramine (TSAA) and Tris(N-salicylidene anthraquinoylamine) (TSAQ) is similar to the report[3].

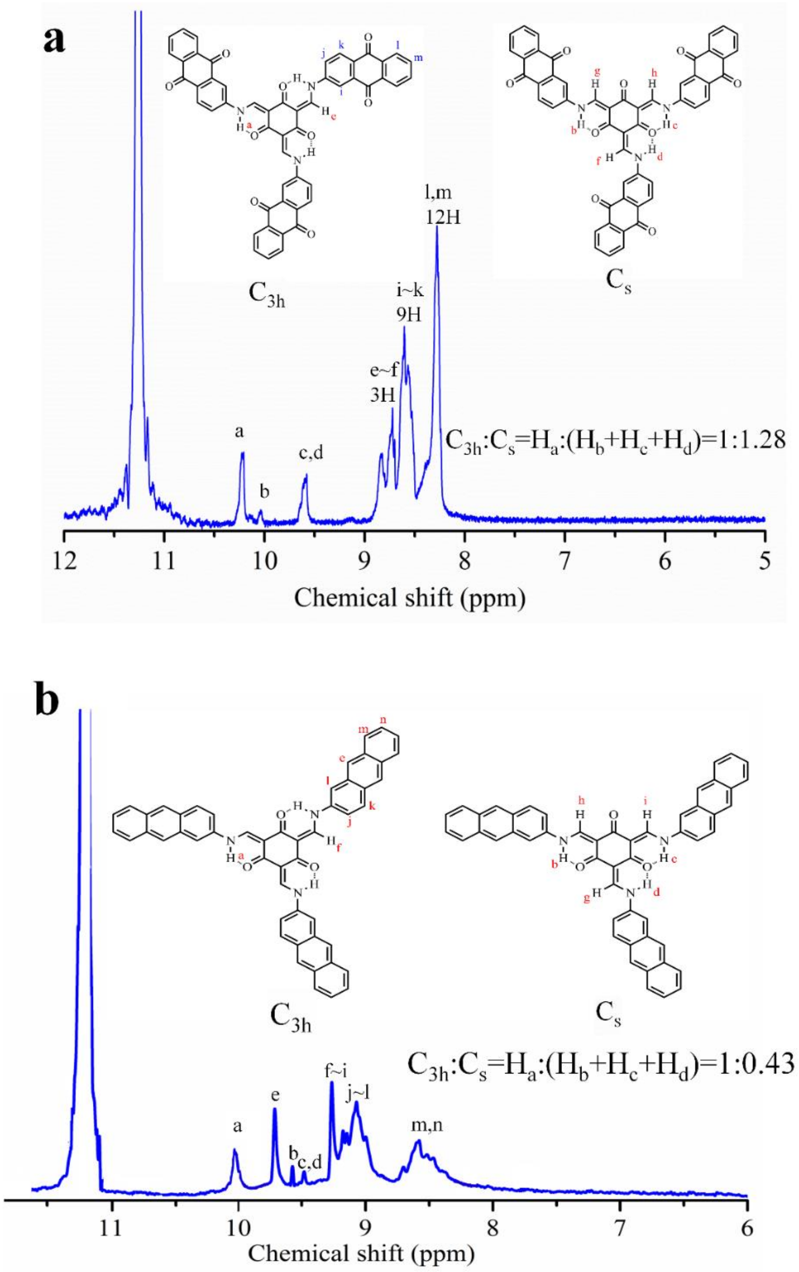

Supplementary Figure 3: Chemical structure by solvent NMR characterization. ^{1}H NMR spectra using D_2SO_4 as a solvent for TSAA (a) and TSAQ (b).

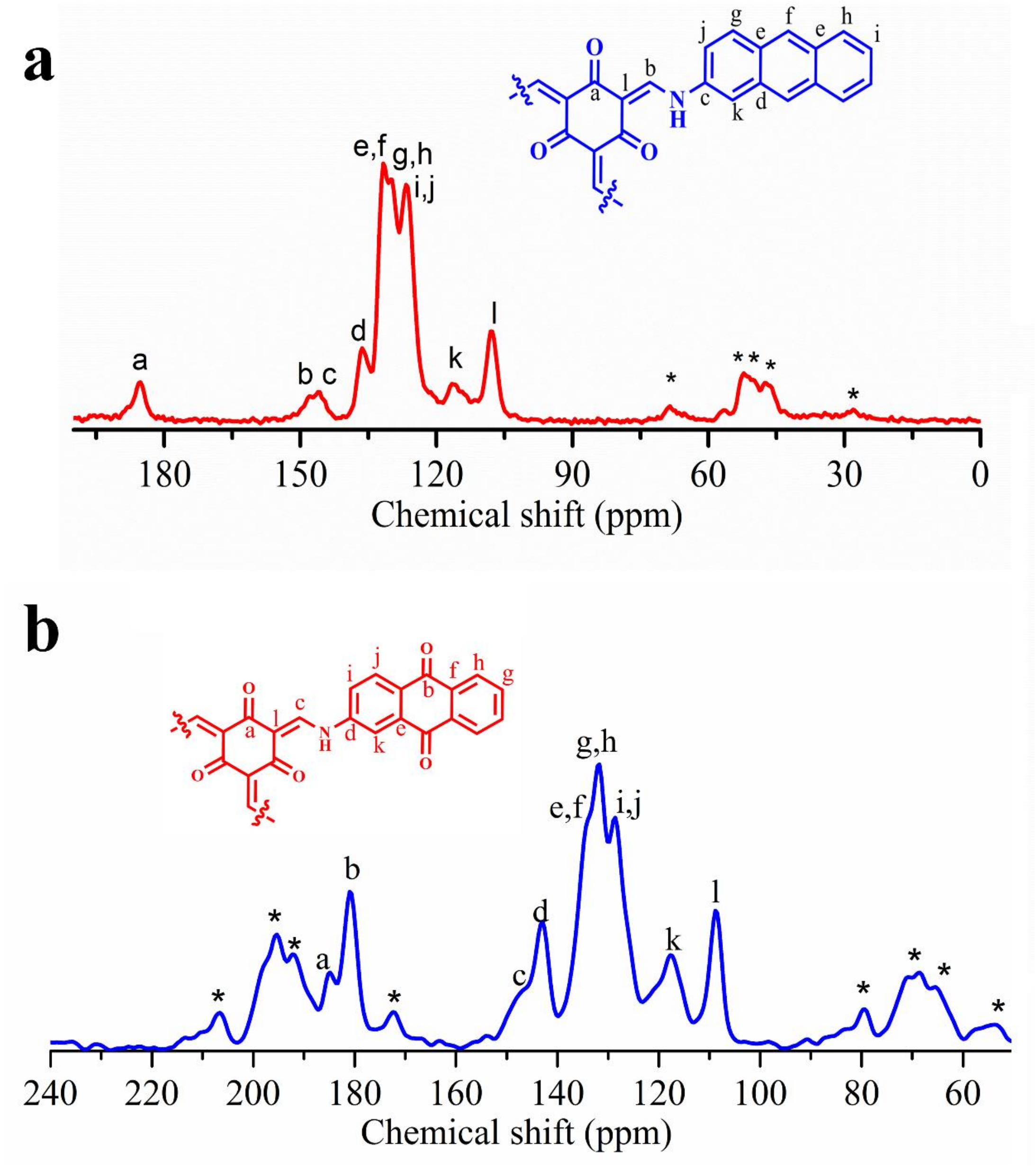

Supplementary Figure 4: Chemical structure by solid state NMR characterization. ^{13}C NMR spectra of TSAA (left) and TSAQ (right). The peaks marked with (*) represent the spinning side band.

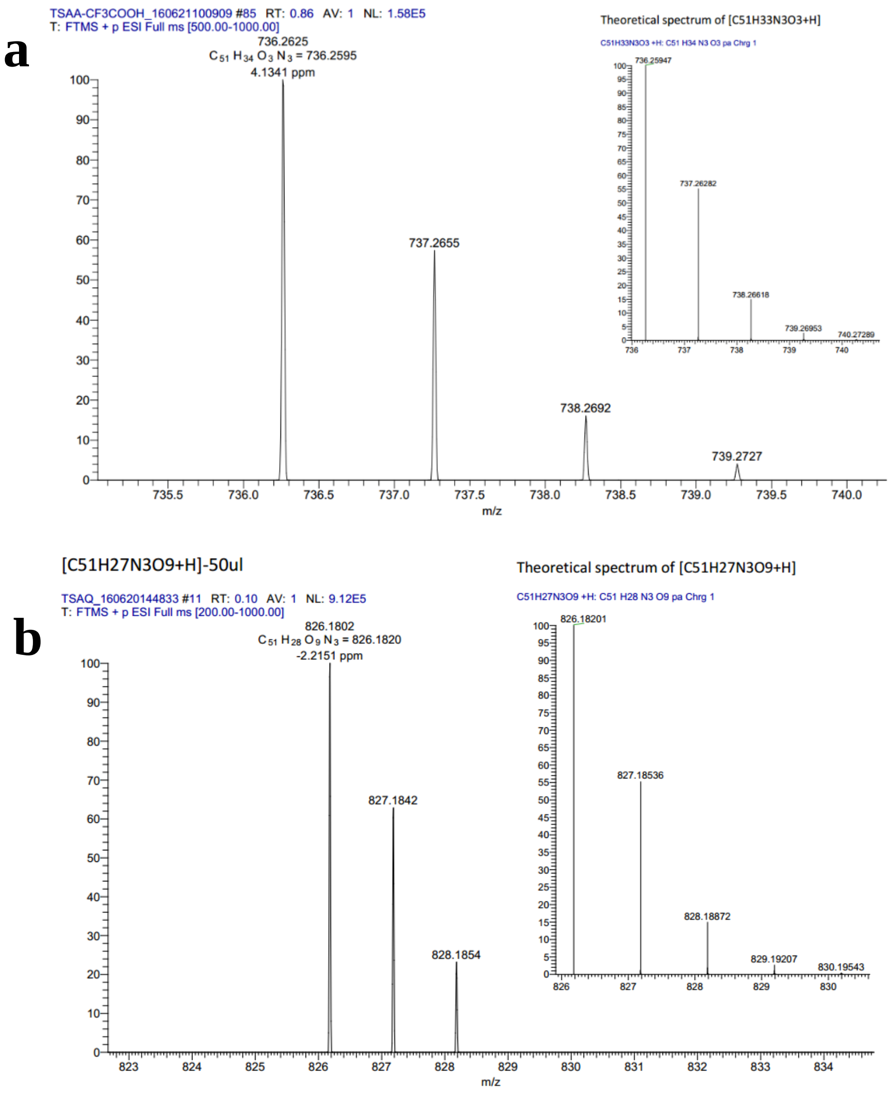

Supplementary Figure 5: The analysis of element composite of the molecules. High resolution mass spectrum of TSAA (a) and TSAQ (b).

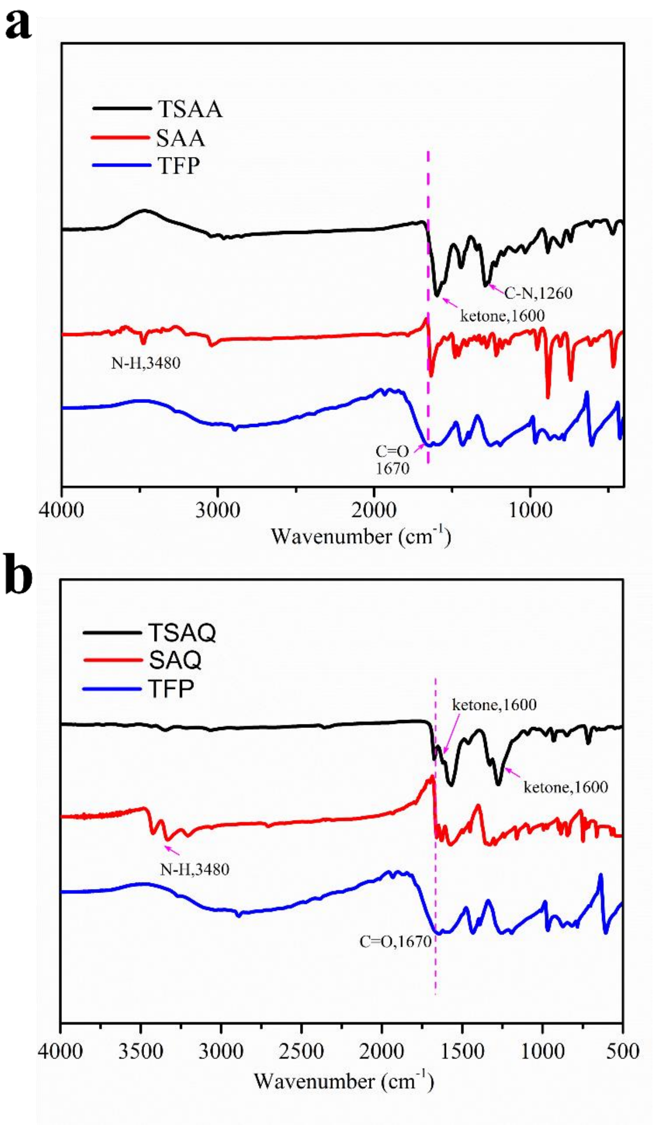

Supplementary Figure 6: The analysis of functional group of the molecules. (a) FT-IR spectra of TSAA and the raw materials, SAA and TFP and (b) spectra of TSAQ and the raw materials, SAQ and TFP. The strong peak at 1670cm^{-1} ascribing to aldehyde group (-HC=O) of TFP was completely disappeared, which imply a total reaction between aldehyde group and anthryl amine.

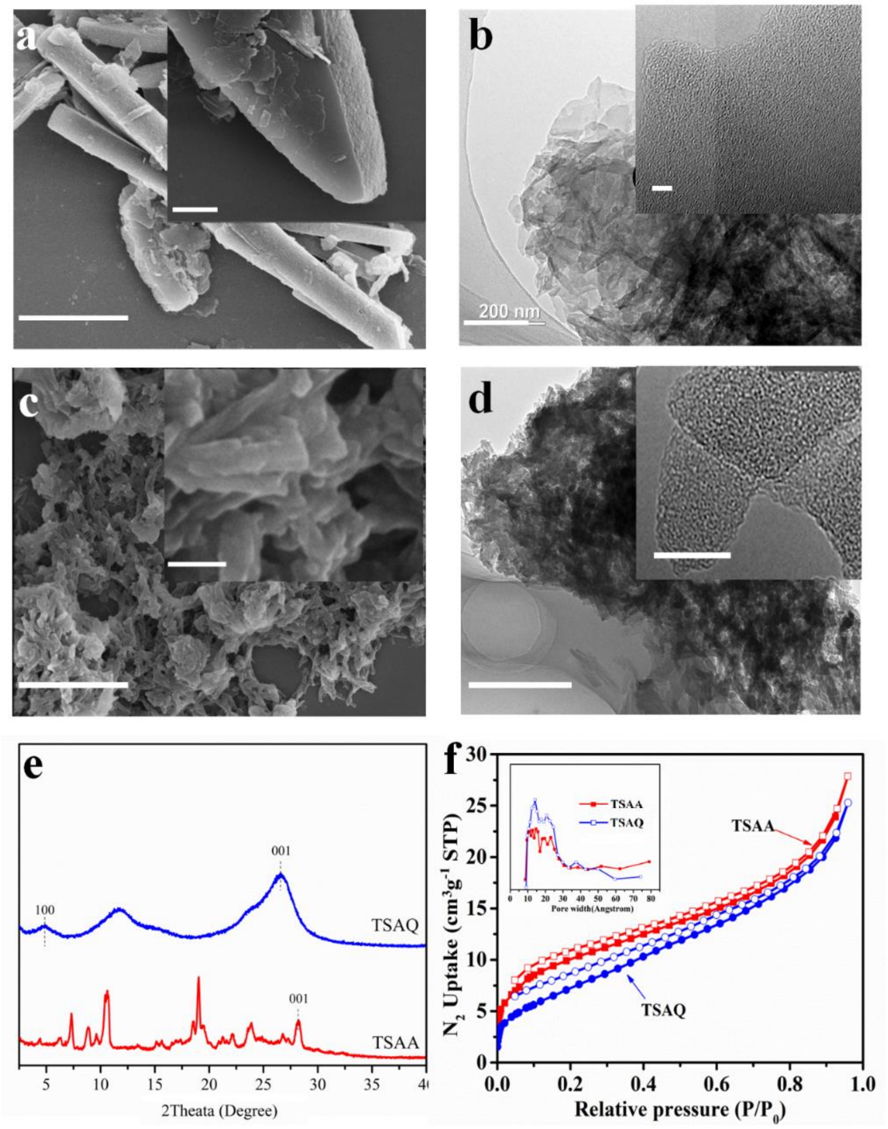

Supplementary Figure 7: Morphology study of TSAA and TSAQ. SEM (a) and TEM (b) images of the TSAA. Insert in (a, b) shows the high-magnification SEM/TEM image of the sample; SEM (a) and TEM (b)images of the TSAQ. Insert in (c, d) shows the high-magnification SEM/TEM image of the sample; (e) PXRD pattern of for TSAA and TSAQ. (f) N_2 adsorption (filled symbols) and desorption (empty symbols) isotherms (left) at 77 K and pore size distributions (insert f) for TSAA and TSAQ. Scale bar: (a) 2μm; (insert a) 500nm; (b) 200nm (insert b) 5nm. (c) 2μm; (insert c) 400nm; (d) 500nm; (insert d) 20nm.

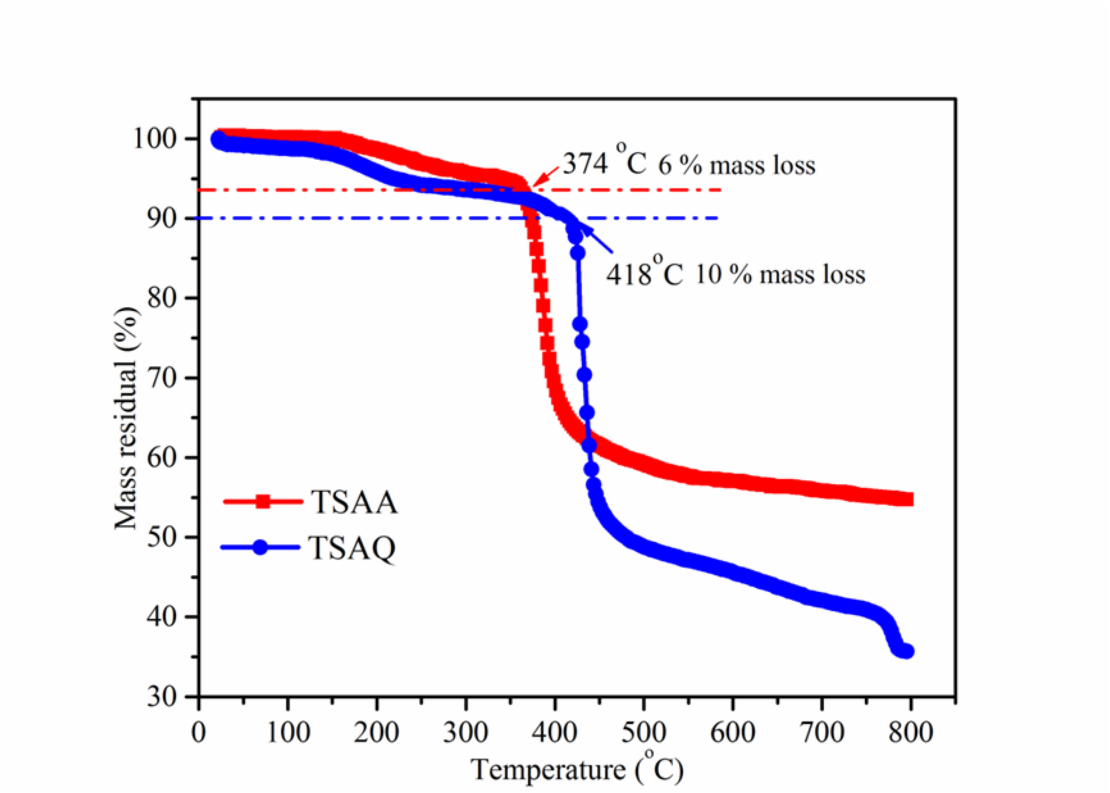

Supplementary Figure 8: Thermal stability study of the electrodes. TGA data of TSAA (red) and TSAQ (blue) under N_2 atmosphere. 6 % mass loss temperature is as high as 374 °C for TSAA while 10 % mass loss temperature is about 418 °C for TSAQ.

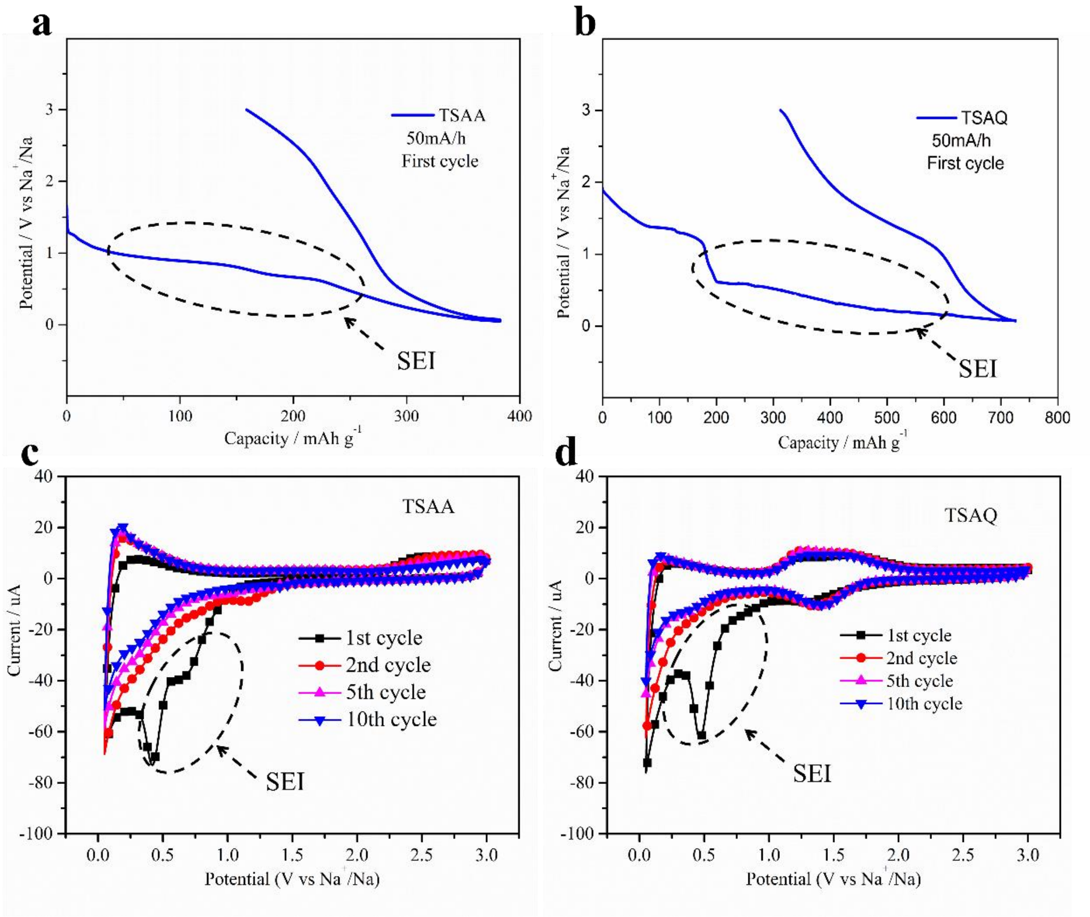

Supplementary Figure 9: The analysis of Coulombic efficiency of the initial cycle. The 1st galvanostatic discharge-charge curves of the TSAA (a) and TSAQ (b) at a current density of 50 mA g^{-1}; The 1st, 2nd, 5th and 10th cycle of cyclic voltammogram (CV) for TSAA (c) and TSAQ (d).

Cyclic voltammogram (CV) curves were tested between 0.05 and 3.0 V (Vs. Na^+/Na) at a scan rate of 0.1 mV s^{-1} under ambient temperature (cal. 25°C) with a half cell within a sodium metal. It is apparent that the CV curve of the first anodic process is quite different from those of subsequent cycles. The well-defined peaks centered at about 0.5 V (Vs. Na^+/Na) only appear in the first CV cycle can be ascribed to the occurrence of some irreversible reactions via the formation of SEI film. The same phenomenon can also be seen from the exclusive plateau ranging from 0.8 to 0.4 V of the TSAA and TSAQ first discharge curve. Comparing the curves of TSAA and TSAQ, the only difference limited at the intensive peak at about 1.5 V (vs. Na^+/Na), which could be attributable to addition of sodium-ion into carbonyl on the anthraquinone group. Both the discharge processes completed with a sharp current declination from 0.7 V to 0.05 V could be ascribed to the formation of the bonding between Na^+ and C atoms according to reports. During the cathodic scans, TSAA displays two oxydic peaks at 0.1 V and 2.4 V while TSAQ shows three peaks at 0.1 V, 1.2 V and 1.6 V. The broad peak around 0.2 V can be ascribed to extraction of those Na-ions "absorbing" by C atoms, while the oxidation of C-O-Na contributes mainly to the oxydic peak above 1.0 V. From the second cycle, the CV profile almost overlaps demonstrating a superior electronic reversibility.

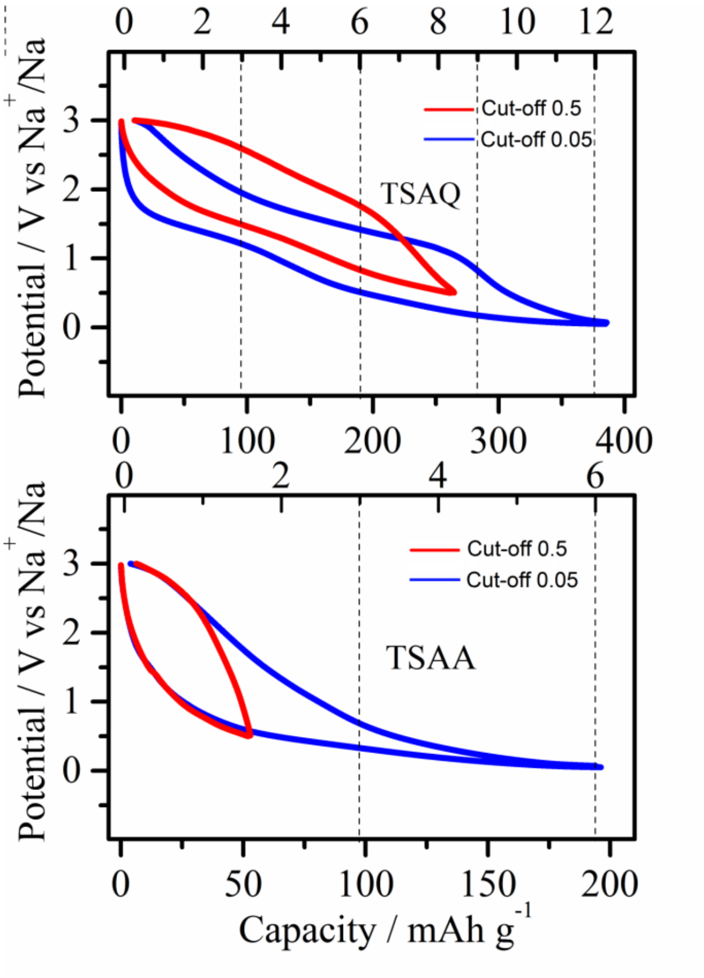

Supplementary Figure 10: The analysis of electro-polarization of the radical carbon. The galvanostatic discharge-charge voltage profiles of the TSAA and TSAQ electrodes with cut-off voltages of 0.5 and 0.05 V. Here we only show the profiles of the 5th cycle because the capacity became stable after the 5th cycle.

The discharge and charge profiles with a cut-off voltage of 0.05 V and 0.5 V for both samples are displayed in Supplementary Fig. 10. We can see that the plateaus of the charge and discharge profiles above 0.5 V mainly contributed from the sodium-ion insertion on the carbonyl groups show an obvious hysteresis. The slopping range below 0.5 V linked to the sodium-ion insertion into radical also displays apparent polarization but much less than that of the carbonyls. As a result, the Na^+ insertion/extraction on the α-C radical displays less polarization compared to that into carbonyl, which indicates that the sodiation/desodiation of the radical intermediates is highly reversible.

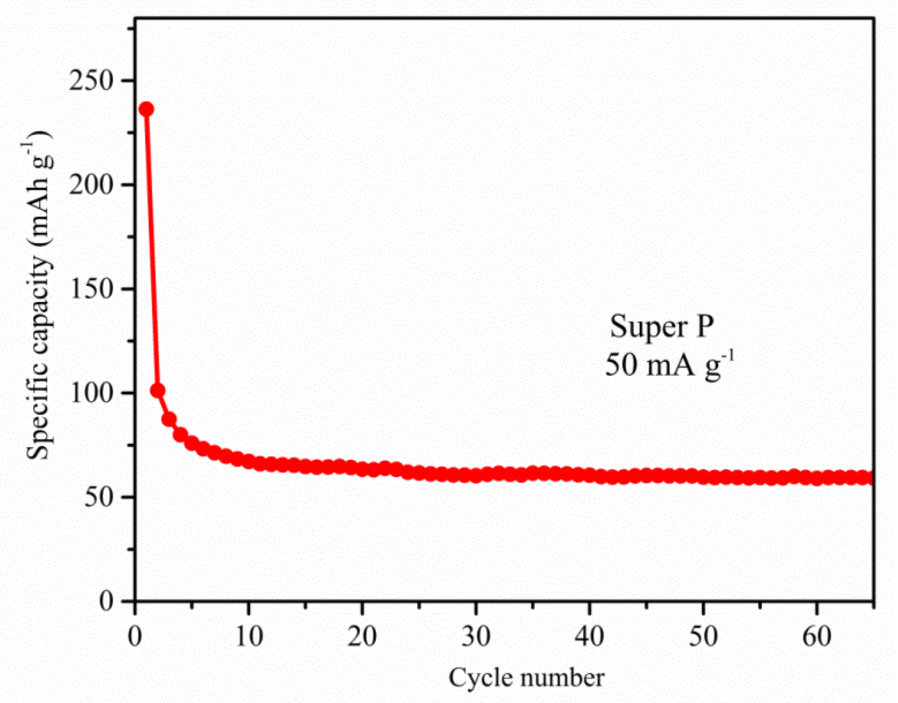

Supplementary Figure 11: The analysis of capacity contribution by Super P. Cycle performance of Super P (0.8 mg cm^{-2}) at a rate of 50 mA g^{-1}.

Since 30 wt% super P as conducting active was also involved in the electrochemical sodiation, this capacity contribution has to be taken into account. As shown in Supplementary Fig. 11, super P demonstrates an overall capacity of 58 mAh g^{-1}, corresponding to a real capacity of 29 mAh g^{-1} (=58 mA h g^{-1}*30 wt% of Super P/60 wt% of active material) in the current electrodes. Therefore, the electrode delivers actual capacity of about 172 and 366 mAh g^{-1}, corresponding to about 5.5 and 11.5 Na^+ ions per molecule can be inserted into the TSAA and TSAQ, respectively.

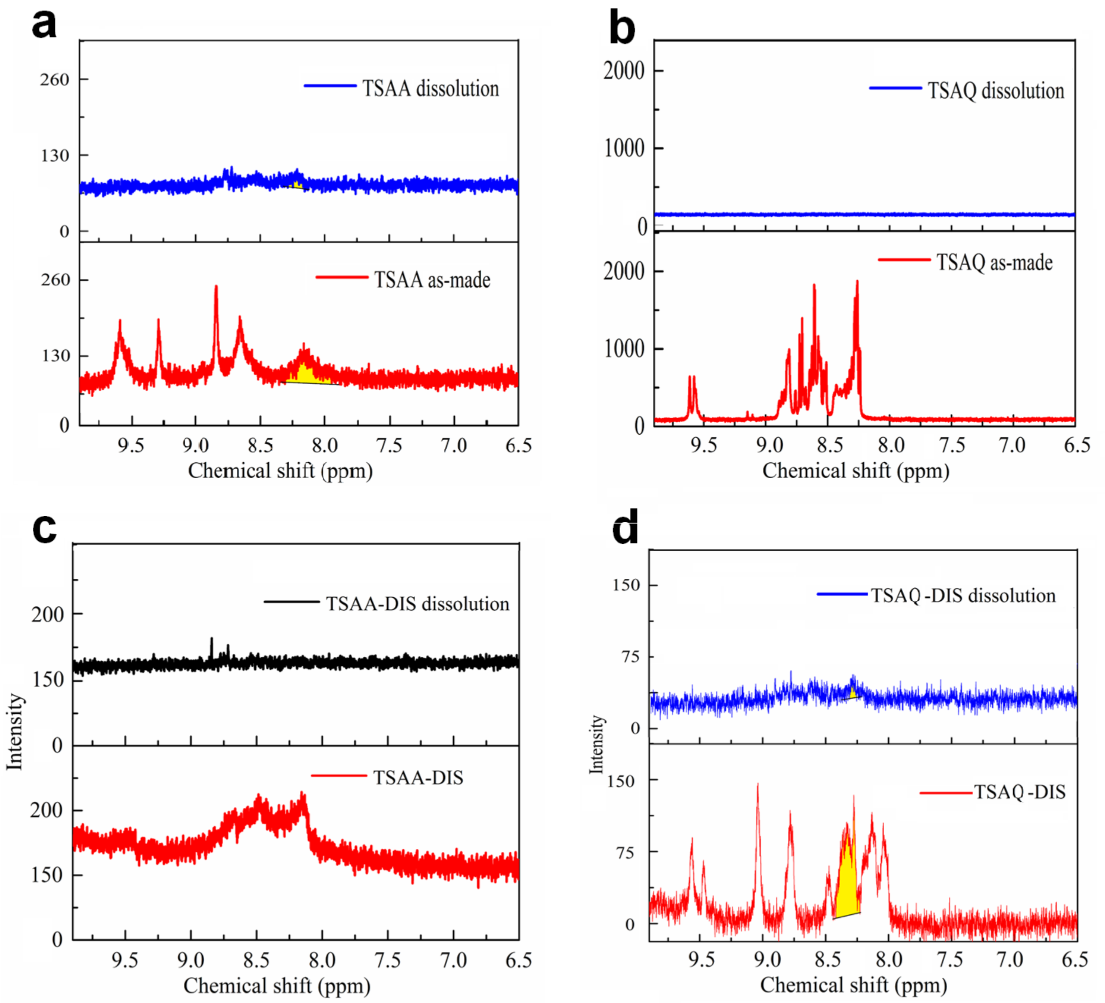

Supplementary Figure 12: The study of the solubility of as-made and sodiated electrodes. (a) ^{1}H NMR spectra using D_2SO_4 as solvent for the pristine TSAA electrode (a, 0.4 mg mL^{-1}) and TSAQ (b, 2.2 mg mL^{-1}) as well as the sample recovered from the electrolyte after the electrodes has been soaked in 5 mL electrolyte for 3 days, respectively. ^{1}H NMR spectra using D_2SO_4 as solvent for the TSAA electrode (c, 0.4 mg mL^{-1}) and TSAQ (d, 0.4 mg mL^{-1}) at fully discharged stage and the sample recovered from the electrolyte after the electrode has been soaked in 5 mL electrolyte for 3 days, respectively. The solubility of the sodiated TSAQ electrode in electrolyte is calculated about 2.2*10^{-3} mg mL^{-1}.

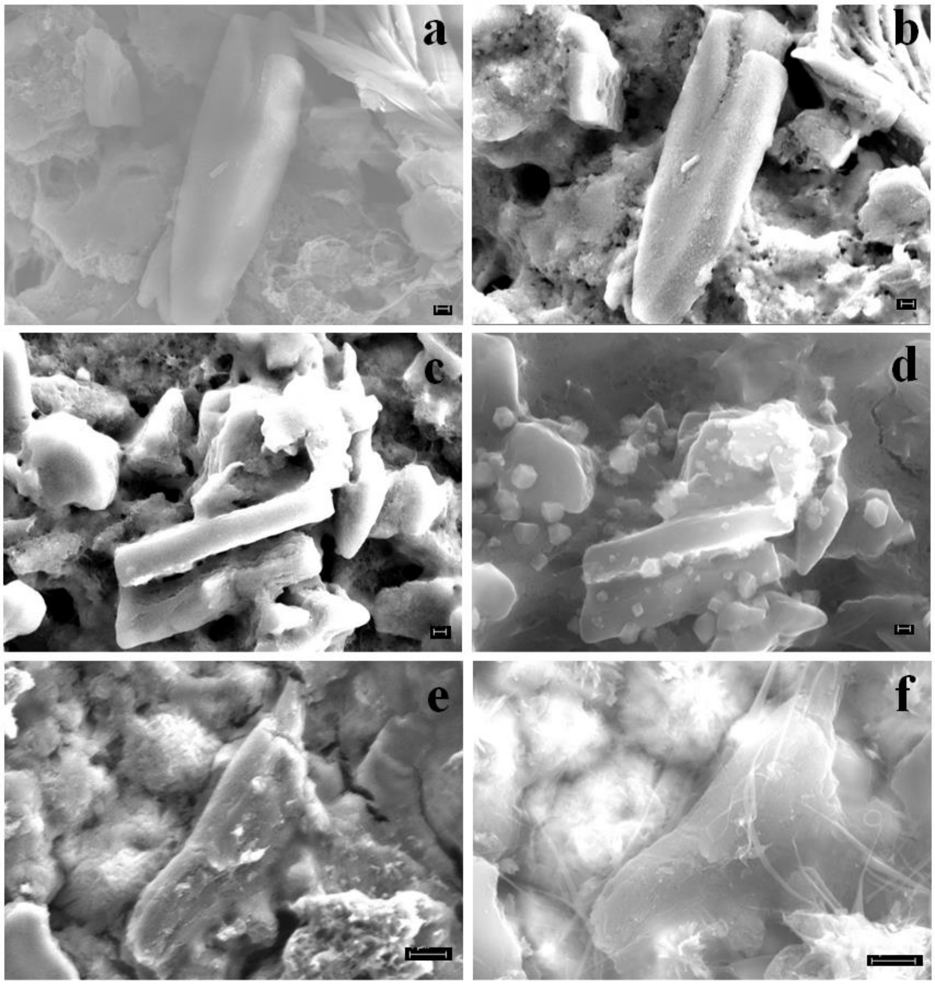

Supplementary Figure 13: The study of dissolubility of the electrodes by SEM. SEM images of the pristine TSAA electrode, (a) before, and (b) after soaked in 5 mL electrolyte for 20 h; SEM image of the TSAA electrode in fully discharged state (c) before and (d) after soaked in 5 mL electrolyte for 20 h. SEM image of the TSAQ electrode in a fully discharged state (e) before and (f) after soaked in 5 mL electrolyte for 20 h. Scale bar: (a) 200 nm; (b) 200 nm; (c) 200 nm; (d) 200 nm; (e) 2μm; (e) 2 μm.

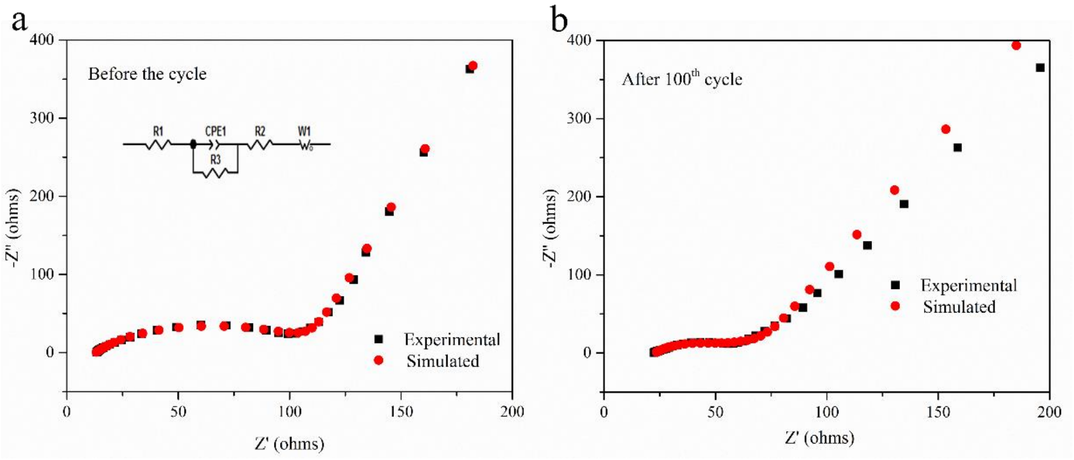

Supplementary Figure 14: The charge transferring and ion immigration property of TSAQ electrode. Typical Nyquist plot of TSAQ half-cell before the cycle (a) as well as after activation at a low current density for 100 cycles (b) and their equivalent circuit, R_1= 12 Ω, R_2=73 Ω, R_3=107 Ω, T_{CPE1}=8*10^{-5} F for (a), R_1= 21 Ω, R_2=0.8 Ω, R_3=22 Ω, T_{CPE1}=8 *10^{-5} F for (b)

The Nyquist plot was further used to reflect the electronic conductivity of TSAQ electrode. It is obvious that a semicircle at medium frequency region. In this plot, the semicircle, corresponding to sodium ions passing through the SEI film and charge transfer between electrolyte and active material, is rigidly consistent with the simulated impedance data. Before cycle, the resistance value of R_{ele} and R_{ct} is around (12 Ω) and (107 Ω), respectively, evidently comparable to reported charge transfer resistance for organic NIBs. After activation for 100 cycles, the resistance value of R_{ele} increased to around 21 Ω and the R_{ct} decreased to 22 Ω. Since R_{ele} represents electronic contact between the active materials and current collector, and the electron transport throughout the electrode, the raising of R_{ele} might be due to the worsening contact between the active particles after long cycle. But the values of 21Ω for R_{ele} and 22 Ω for R_{ct} are pretty low, indicating fast charge transport and high rate performance.

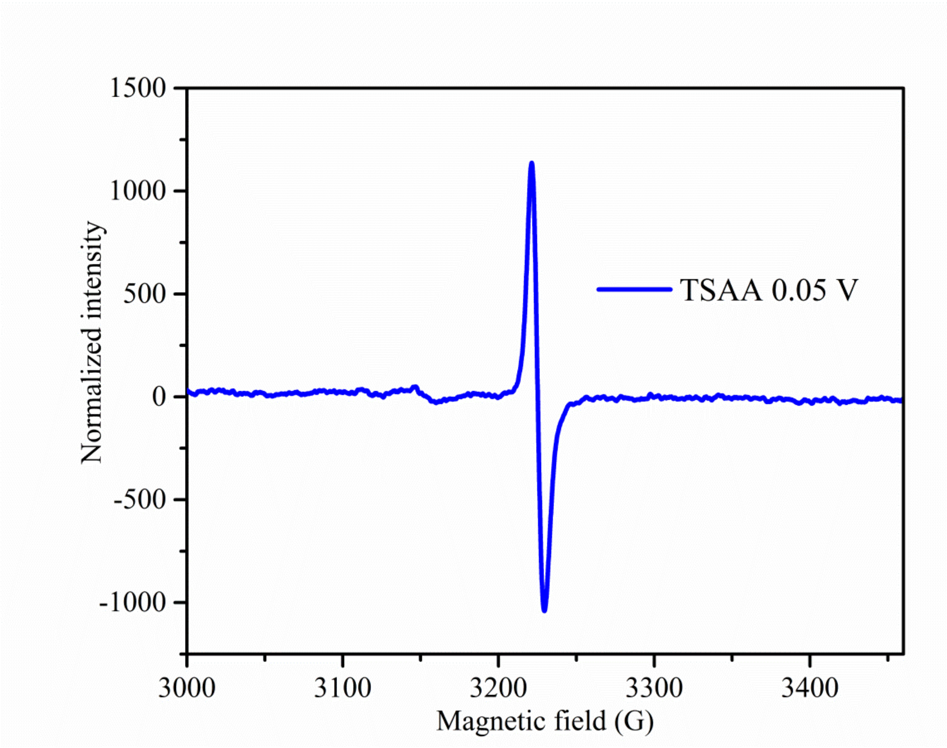

Supplementary Figure 15: The analysis of unpaired electron for TSAA. CW-EPR for TSAA electrode at 0.05 V.

a b

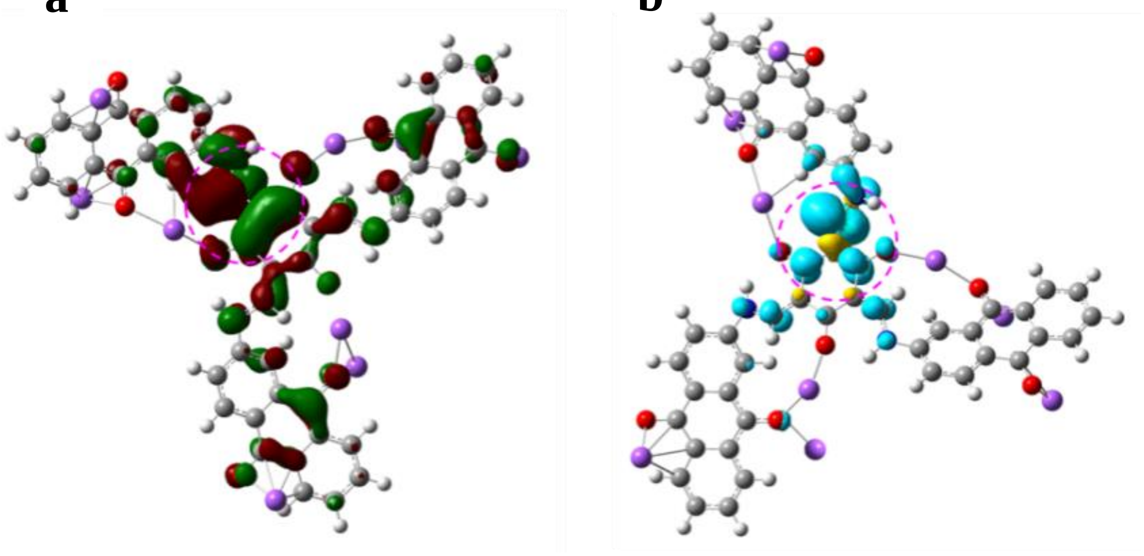

Supplementary Figure 16: The simulation analysis of radical location of the sodium-insertion state. Calculated SOMO (a) and spin density distribution (b) of radical intermediate TSAQ-9Na (B3LYP/6-31G).

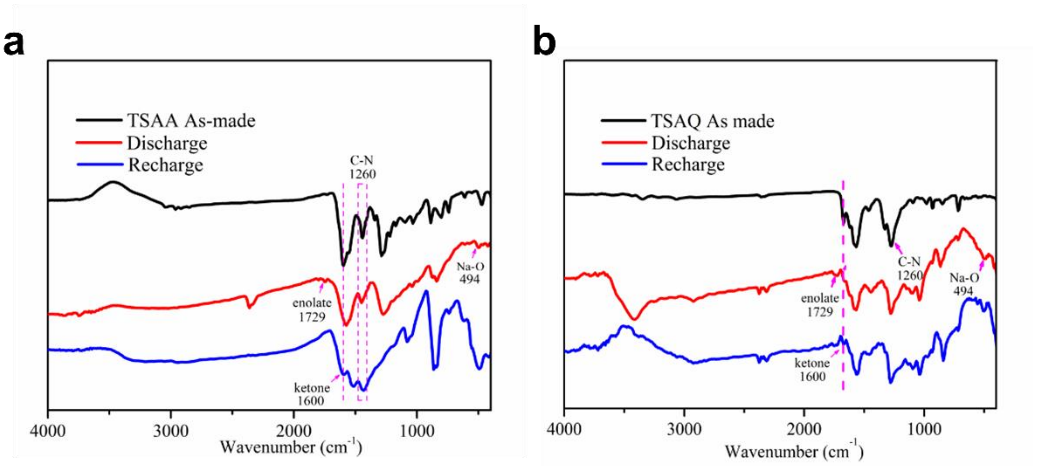

Supplementary Figure 17: The analysis of redox process of carbonyl groups of the electrodes. FT-IR spectra for the different states of TSAA (a) and TSAQ (b): as-made (Black), the fully discharge to 0.05 V state (Red) and the fully recharge state to 3.0 V (Blue).

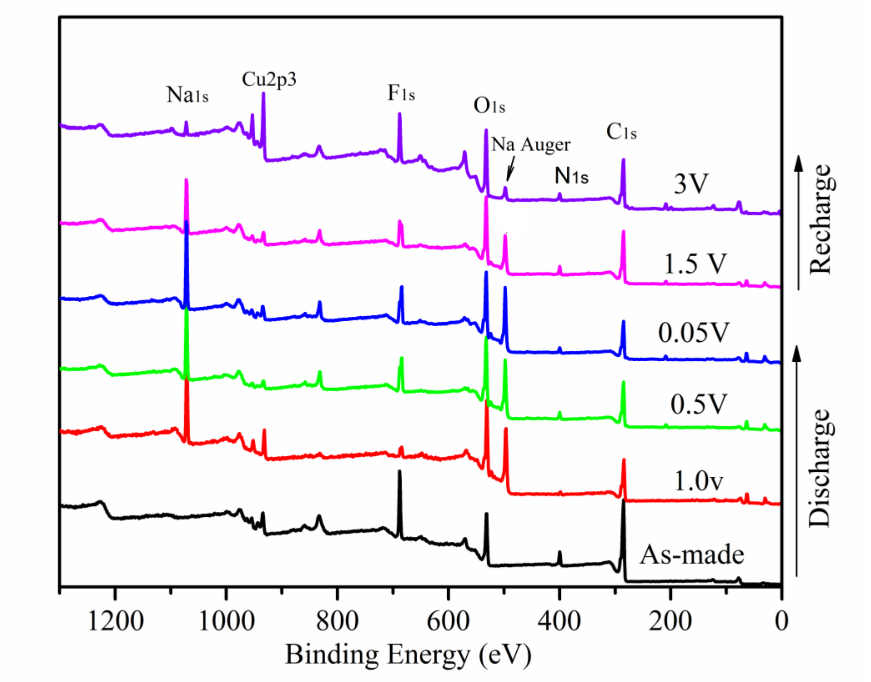

Supplementary Figure 18: The investigation of the changes of element valence at different stages. Full X-ray photoelectron spectroscopy (XPS) spectra for TSAQ electrode at different states: as-made, discharge to 1.0 V, discharge to 0.5 V, discharge to 0.05 V, recharge to 1.5 V and fully recharge to 3 V.

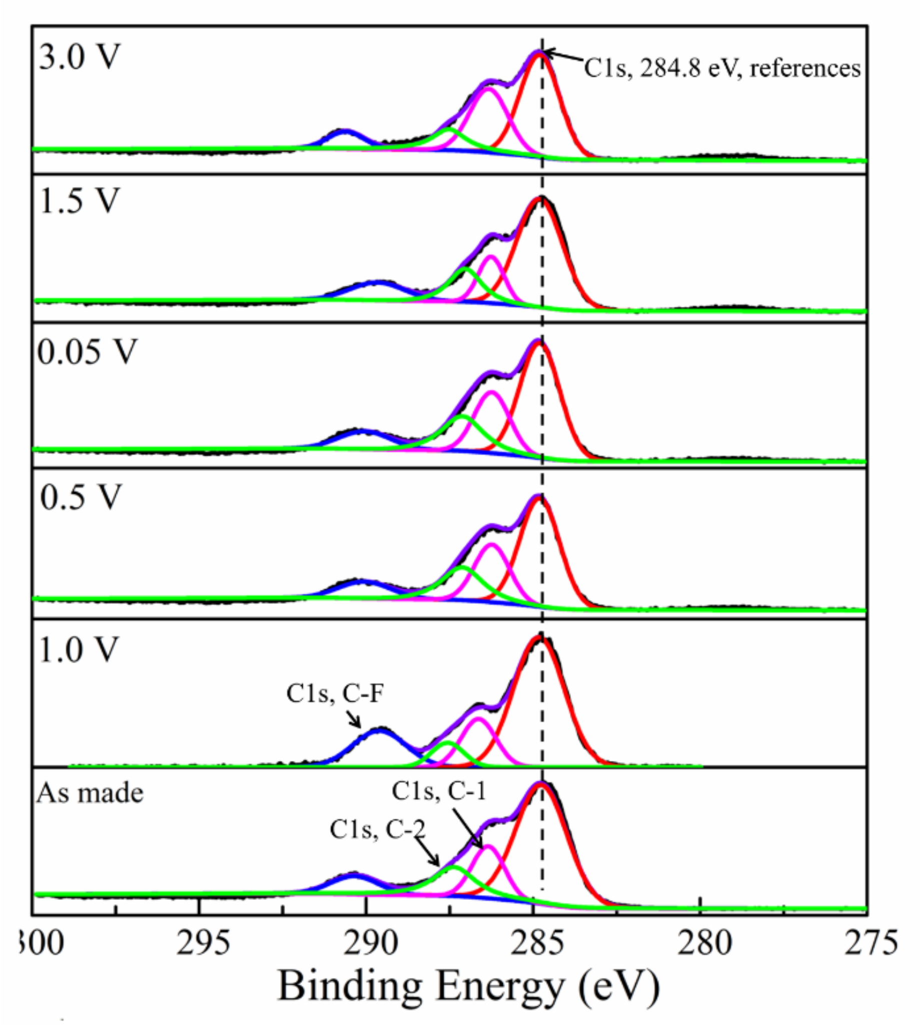

Supplementary Figure 19: The analysis of the revolution of carbon valence at different dis-/re-charge stages. C_{1s} XPS spectra of TSAQ electrode at different states: as-made, discharge to 1.0 V, discharge to 0.5 V, discharge to 0.05 V, recharge to 1.5 V and fully recharge to 3 V. The binding energies were referenced to C1s line at 284.8 eV (+/- 0.1 eV) from graphite carbon. The peak C-1 refers to the XPS signal of the Carbon in C=O groups of quinone structure, and C-2 represents those in C=O groups of β-Keto.

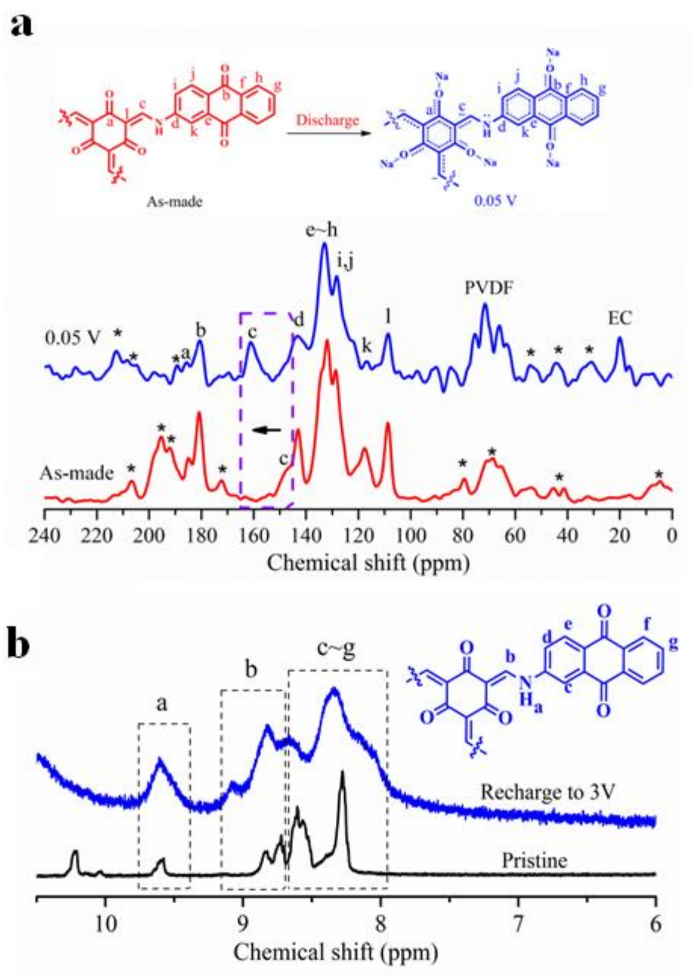

Supplementary Figure 20: The analysis of the resonance effect toward α-C. (a) Solid state ^{13}C NMR spectra of the TSAQ electrode at different states: as-made and discharge to 0.05 V. (b) ^{1}H NMR spectra of the TSAQ electrode at different states: as-made and fully recharge to 3 V.

The ^{13}C signal at about 146.8 ppm of the pristine material rightly shifts to a higher chemical shift about 161.2 ppm after discharged to 0.05 V. Such a high chemical shift of the α-C implies strong resonance effect coming from the neighboring aromatic substitution (benzene and arylamines) and thus a conjugated structure of the sodiated state. The strong shielding effect of the π system of the TSAQ-12 Na has contributed to the high chemical shift. All the ex-situ ^{1}H NMR, FT-IR, XANES, XPS and ESR spectra indicate a highly reversible electro-chemical transformation of TSAQ.

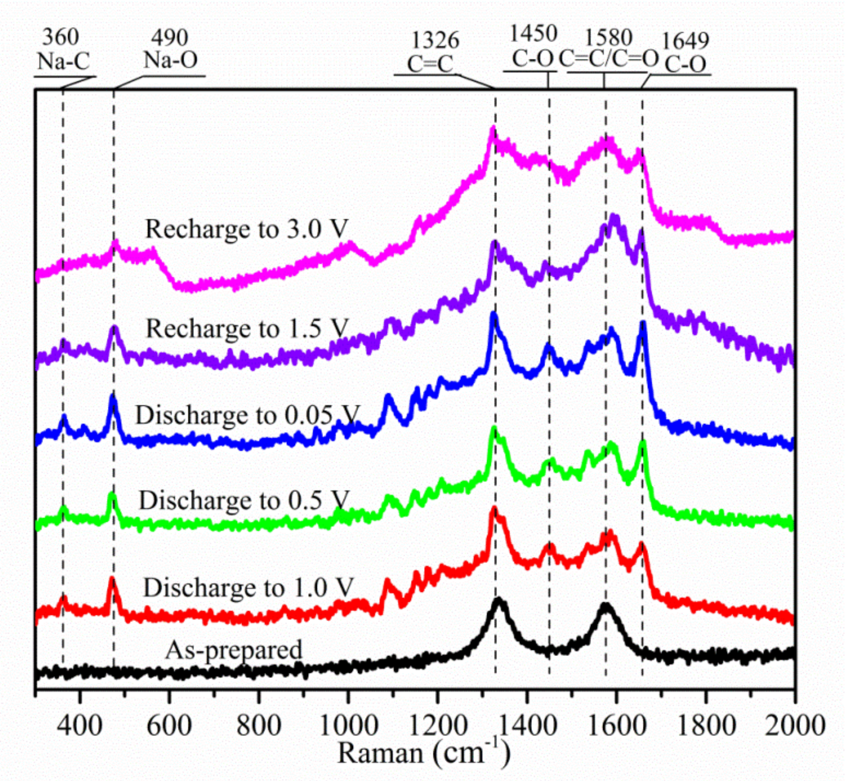

Supplementary Figure 21: The investigation of C-Na interaction during the dis-/re-charge process. Ex-situ Raman spectra of the TSAQ electrode at different states: as-made, discharge to 1.0 V, discharge to 0.5 V, discharge to 0.05 V, recharge to 1.5 V and fully recharge to 3 V. The Raman spectra show almost the same transformation as FT-IR displays. It is especially worth noting that the spectra illustrate the reversible reaction between sodium and carbon (e.g. Na-C bonding) at 360 cm^{-1}.

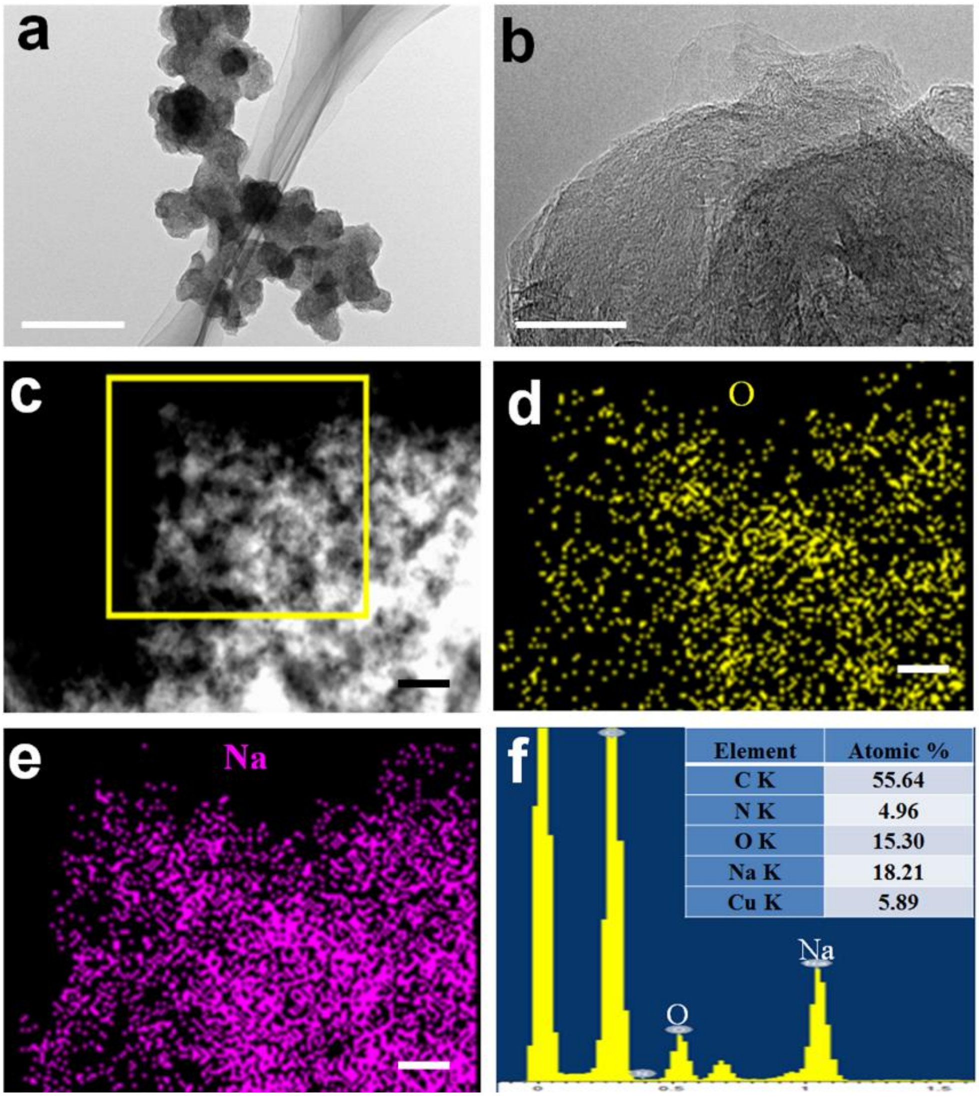

Element	Atomic %
C K	55.64
N K	4.96
O K	15.30
Na K	18.21
Cu K	5.89

Supplementary Figure 22: The morphology study of the electrodes after fully discharged. TEM image of the electrode 100 cycles (a); HR-TEM image of the electrode after 100 cycles (b). TEM images (c) of the TSAQ electrode in fully discharge state and the corresponding EDX elemental mapping of oxygen (d), sodium (e) and the corresponding EDX full spectrum (Na/O=1.19) (f), showing the homogeneous distribution of sodium and oxygen element. Scale bar, a, 500nm; b, 20nm; c, 20 nm; d and e, 10nm;

Supplementary Table 1: Summary of electronic and thermal free energies (Hartree) of optimized structures calculated at the B3LYP/6-31G level.

Compound	TSAQ	TSAQ-6Na	TSAQ-9Na	TSAQ-12Na
$G^{ø}m$ (Hartree)	-2801.049	-3775.068	-4262.016	-4748.940

$G^{ø}m$, Na(0)=-162.2950 Hartree. Using these data, the redox potential of TSAQ/ TSAQ-6Na, TSAQ-6Na/TSAQ-9Na and TSAQ-9Na/TSAQ-12Na is calculated 1.12 V, 0.58 V and 0.36 V according to the equation nFE=-ΔG, respectively. These values are close to the electrochemically determined average potentials of the corresponding electrode.

Supplementary Note 1: Discussion of the solubility

We assume that there is solubility equilibrium of the radical intermediate (R·) in charged states as the following equation shows.

$$R\cdot(s) \rightleftharpoons R\cdot(l) \longrightarrow R\text{-}R$$

R·(s) means the solid state radical intermediates that fixed on the copper foil, while R·(l) represents those dissolved into the electrolyte. And R-R displays the dimer linked to the combination of irreversible radical side-reactions. It is certain that R·(l) has a higher potential to form R-R than R·(s) does because of a higher probability of molecule collision at a solution state. In return, the excessive formation of R-R will drive the dissolving reaction to the right side. As a consequence, a large amount of R·(s) will finally transform to the inactive materials and thus result in a capacity degradation as DCCA displays. And as we have discussed in the manuscript, the high reactivity toward to the inactive R-R of the radical intermediates of R·(s) and R·(l) could be effectively restricted by the resonance effect of the p-π conjunction and the generated 9-π-electron aromatic Hückel ring and hindrance effect of the rigid geometry of the generated TSAQ-9Na or TSAA-9Na radical intermediates.

The π-conjugated systems of TSAA and TSAQ result in a layer-by-layer molecular arrangement through the intermolecular interactions (e.g. π–π or C-H•••π interactions), which makes them hardly soluble in solvent such as DMC and EC. The materials TSAQ and TSAA displayed a very poor solubility in common solvents with high polarity or non-polarity like DMSO, NMP, $CHCl_3$, ethanol as well as the electrolyte solvents.

In order to collect the solubility information of the electrodes in electrolyte, lots of measurements such as ICP, spectrophotometer UV, liquid chromatography (LC) have been tentatively utilized. However, both of the electrodes display poor solubility in common solvents like CH_3OH, CH_3CN, DMSO, NMP, $CHCl_3$, DMF and so on, therefore it is really hard to obtain a standard reference to measure the exact amount of the materials in the electrolyte. Fortunately, we have found that the TSAA and TSAQ electrode as well as theirs sodiated electrodes could be easily dissolved by concentrated sulphuric acid (H_2SO_4) and CF_3COOH. It is easy to obtain a quantitative solution of the electrodes by using such solvents. In addition, as an important quantitative analysis method, NMR has an advantage of high accuracy and interference immunity. As a result, the ^1H NMR method was used to verify the solubility of the electrodes by using D_2SO_4 as solvent.

The ^1H NMR spectra of the TSAA and TSAQ pristine material were collected by using D_2SO_4 as solvent with a concentration of 0.4 mg mL^{-1} for TSAA, and 2.2 mg mL^{-1} for TSAQ. The two electrodes were soaked in 5 mL electrolyte (EC: DMC=1:1) for three days. The remaining solid was collected via filtration. The filtrated solution was heated to evaporate the DMC solvent and the residual solid was expected to be a mixture of EC and those TSAQ or TSAA compounds. The residual was completely

dissolved by 0.5 mL D_2SO_4. Then the ^{1}H NMR spectra were collected by using 0.5 mL D_2SO_4 as solvent. Since the signals of EC are only located in around 1~3 ppm of chemical shift while the signals due to TSAA or TSAQ were ranging from 6 to 10 ppm of chemical shift, we can qualitatively analyze the solubility of the organic electrodes. Therefore, by comparing of the integral area of the specific peak, the solubility of the two electrodes in the electrolyte could be obtained.

As can be seen from Supplementary Fig. 12, the spectrum of the TSAA dissolution sample displays a very small peak at the chemical shift of about 8.3 ppm. After calculation, the ration of the integral area is about 1/20, indicating a solubility of about 4 mg mL^{-1}, which value could be negligible. The spectrum of the TSAQ dissolution sample shows almost no proton signal, implying a limited dissolubility of the electrode in electrolyte. Similarly, we can draw the conclusion that both of the sodiated electrodes display very limited solubility in the studied electrolyte solution.

It is understandable that if the organic compounds have significant solubility in the electrolyte, the organic electrodes will experience obvious variations in the morphology. Therefore, we have analyzed the morphology difference of the organic electrodes before and after long time of reaction in the electrolyte by SEM. The SEM images in Supplementary Fig. 13 display little differences after the treatment with 5 mL electrolyte for 20 h, which implies very limited dissolution of the TSAA electrode. As can be seen, except that there are some nano-particles appearing on the surface, the morphology and size of the fully sodiated TSAA electrode were almost unchanged after soaked in electrolyte for very long time. Such nano-particles could probably come from the re-equilibration interaction between the sodiated electrode and electrolyte. It is clear that both the pristine and sodiated TSAA electrodes are stable in the organic electrolyte. The SEM images for the sodiated TSAQ electrode display little differences (Supplementary Fig. 13e and f) before and after treatment with 5 mL electrolyte for 20 h, also implying very limited dissolution.

Supplementary References

1. Chong, J. H., Sauer, M., Patrick, B. O. & MacLachlan, M. J. Highly stable keto-enamine salicylideneanilines. *Org. Lett.* **5**, 3823-3826 (2003).

2. DeBlase, C. R., Silberstein, K. E., Truong, T. T., Abruna, H. D. & Dichtel, W. R. beta-Ketoenamine-linked covalent organic frameworks capable of pseudocapacitive Energy Storage. *J. Am. Chem. Soc.* **135**, 16821-16824 (2013).

3. Yelamaggad, C. V., Achalkumar, A. S., Rao, D. S. S. & Prasad, S. K. A new class of discotic mesogens derived from Tris(N-salicylideneaniline)s existing in C-3h and C-s keto-enamine forms. *J. Org. Chem.* **72**, 8308-8318 (2007).

4. Guerra, M., Jones, D., Distefano, G., & Modelli, A. Electronic structure and XPS shake-up satellites of para-benzoquinones. Chemical physics, 85(3), 389-396 (1984)
